# Tai Chi Exercise and Bone Health in Women at Perimenopausal and Postmenopausal Stages: A Systematic Review and Meta-Analysis

**DOI:** 10.3390/life15111678

**Published:** 2025-10-28

**Authors:** Wenhui Yin, Zhuo Zeng, Wenyan Yin, Long Xi, Dong Wu, Fengjie Qiao

**Affiliations:** 1 Division of Sports Science and Physical Education, Tsinghua University, Beijing 100084, China; yinwh24@mails.tsinghua.edu.cn (W.Y.); xilong282@gmail.com (L.X.); 2 Strength and Conditioning Training College, Beijing Sports University, Beijing 100084, China; 2024210137@bsu.edu.cn; 3 School of Physical Education and Health, East China Normal University, Shanghai 200241, China; yinwenyan921@163.com; 4 School of Chinese Wushu, Beijing Sport University, Beijing 100084, China; wudonglunwen@163.com

**Keywords:** Chinese traditional exercise, bone, menopausal, female, menstruation, meta-analysis

## Abstract

This study systematically examined the effects of Tai Chi exercise on bone health in menopausal women, with subgroup analyses of potential moderators. A systematic search was conducted across nine databases (PubMed, Web of Science, Cochrane Library, EBSCO-Medline, EBSCO-Sportdiscus, Embase, CNKI, VIP and Wanfang Data) on June 1 and updated on 14 September 2025 to identify controlled trials evaluating perimenopausal or postmenopausal women. A three-level meta-analysis was performed to pool effect estimates, reported as standardized mean differences (SMDs), with heterogeneity further explored through subgroup analyses. Across 16 studies involving 1091 participants aged 49–64 years, Tai Chi interventions led to significant improvements in bone health. Training protocols ranged from 6 to 104 weeks, with sessions lasting 30 to 90 min. Bone mineral density (BMD) improved significantly at the femoral neck (SMD = 0.50), greater trochanter (SMD = 0.61), and lumbar spine L2–L4 (SMD = 0.81), with stronger effects observed in perimenopausal women. Bone mineral content (BMC) also increased significantly in menopausal women (SMD = 1.63, I^2^ = 91.46%), although heterogeneity was substantial, and no significant differences were detected in subgroup moderators. In contrast, no significant effects were found for bone mineral metabolism (*p* = 0.38) or bone turnover markers (*p* = 0.25). According to GRADE assessments, the certainty of evidence ranged from low to moderate across these outcomes. In conclusion, while Tai Chi has been shown to improve BMD and BMC in menopausal women, the relatively high heterogeneity observed for BMC necessitates cautious interpretation of these particular outcomes. In contrast, no statistically significant effects were observed on bone mineral metabolism (BMM) and bone turnover markers (BTMs). Notably, given the significant differences observed between perimenopausal and postmenopausal women, future well-designed studies that stratify participants by menopausal status and possess adequate statistical power are needed to further explore the potential differential effects of Tai Chi on bone health.

## 1. Introduction

Osteoporosis can occur at any age. It is characterized by fragile and brittle bones, but it is more prevalent among postmenopausal women [1]. The perimenopause, which is also called the menopausal transition, is a time period during which middle-aged women experience irregular menstruation and intermittent amenorrhea as they progress into menopause [2]. This stage is characterized by substantial hormonal fluctuations [3]. During this period, women undergo changes in bone structure, with BMD declining rapidly, thereby increasing the risk of osteoporosis. After menopause, the rate of BMD loss slows down but continues persistently. In a study involving 1038 women [4], reported that each year after the final menstrual period was associated with an annual decrease of 0.006 g/cm^2^ in lumbar spine BMD and 0.004 g/cm^2^ in femoral neck BMD. The 10-year cumulative loss of BMD was 10.6% at the lumbar spine and 9.1% at the femoral neck [5]. Low BMD is one of the most important determinants of fracture risk [6]. It has been reported that approximately 30–40% of postmenopausal women have osteoporosis or low bone mass [7,8], and more than 30% of these women experience at least one fracture [9,10]. Virtually all types of fractures are associated with an increased risk of premature death [10]. Therefore, identifying effective interventions to promote skeletal health in perimenopausal and postmenopausal women is essential for improving women’s quality of life and long-term health worldwide.

Evidence has confirmed that human bone density can be improved through appropriate physical exercise. Exercise causes the contraction of muscle groups and resulting stresses on the bones, inducing hormonal changes that affect bone metabolism [11,12]. In recent years, tai chi has attracted growing attention because of its potential health benefits, safety, low cost, and increasing popularity. It has become an alternative form of exercise that is increasingly embraced in Western countries [13,14]. Several randomized trials have demonstrated that tai chi can effectively improve lumbar spine BMD (L2–4) in perimenopausal women, with improvements of approximately 5.05%, as reported in a previous study [15], enhance estrogen levels and bone metabolic markers [16], and help slow bone loss in weight-bearing sites among postmenopausal women [17]. However, other studies have reported that the beneficial effects of tai chi on BMD are limited, with no convincing evidence supporting its role in improving bone mass [18,19]. In this context, a systematic evaluation is needed to assess whether tai chi is effective in maintaining skeletal health among perimenopausal and postmenopausal women.

Nevertheless, significant gaps remain in the current evidence base regarding the effects of tai chi on women’s bone health. On the one hand, some published meta-analyses have included only perimenopausal [20] or only postmenopausal women [21], whereas others have combined both groups without distinguishing between their physiological characteristics [22,23]. On the other hand, many randomized controlled trials (RCTs) have not clearly defined participants’ reproductive stage, limiting the specificity and comparability of their findings. It is noteworthy that perimenopausal and postmenopausal women differ substantially in bone metabolism. Perimenopause is a particularly vulnerable period for bone loss [24], during which bone density declines rapidly, whereas bone loss continues more gradually after menopause [25,26]. In addition, hormonal profiles and bone metabolism differ between the two stages [27]. Therefore, whether tai chi exerts comparable effects on skeletal health in perimenopausal versus postmenopausal women remains unclear. With the growing availability of high-quality RCTs in recent years, synthesizing the latest evidence has become essential. Therefore, systematic reviews and meta-analyses are now needed to separately evaluate the effects of tai chi on bone health in these two populations. Such efforts will provide more robust and stage-specific evidence to guide precision exercise interventions for women’s skeletal health.

## 2. Materials and Methods

This systematic review was conducted in accordance with the Preferred Reporting Items for Systematic Reviews and Meta-Analyses (PRISMA 2020) guidelines [28]. The completed PRISMA 2020 checklist is available in Appendix A. The review protocol was prospectively registered in the International Prospective Register of Systematic Reviews (PROSPERO; Registration ID: CRD420251128292; date of access: 26 August 2025).

### 2.1. Information Sources and Search Strategy

A systematic literature search was conducted in seven English-language (PubMed, Web of Science, Cochrane Library, MEDLINE, SPORTDiscus, Embase) and three Chinese-language (CNKI, VIP, Wanfang) databases. The initial search was finalized on 1 June 2025 and an updated search was run on 14 September 2025 to capture the most recent publications.

The search strategy was based on the PICOS framework, combining terms for the target population (e.g., “menopause,” “perimenopause”) and the intervention (e.g., “Tai Chi,” “Taijiquan”). To maximize literature retrieval, the primary database search was supplemented by secondary search techniques, including screening the reference lists of included studies and citation tracking. The complete, detailed search strategy is available in Appendix B.

### 2.2. Selection Process

Following the removal of duplicates using EndNote X9 (Clarivate, Philadelphia, PA, USA), the titles and abstracts of all unique records were screened independently by two reviewers (Y.W.H., Z.Z.). This screening was based on the predefined eligibility criteria (see Section 2.3). The same two reviewers then independently assessed the full texts of potentially eligible studies for final inclusion. At both screening stages, any disagreements were resolved through discussion or, if necessary, adjudicated by a third reviewer (Y.W.Y.).

### 2.3. Eligibility Criteria

To more clearly evaluate the independent benefits of Tai Chi exercise on bone health in menopausal women, specifically in comparison to non-exercise or usual care interventions, studies employing high-intensity exercise as a control group were excluded from this meta-analysis. This methodological choice aimed to prevent the introduction of confounding effects and increased heterogeneity that might arise from active intervention control groups, thereby allowing us to better focus on the potential benefits of Tai Chi as a gentle intervention. The detailed inclusion and exclusion criteria were meticulously defined using the PICOS framework (Population, Intervention, Comparison, Outcome, Study design) and are presented in Table 1.

### 2.4. Data Extraction

Data extraction was conducted by the same two reviewers [Y.W.H, Z.Z] who performed the screening, using a customized Microsoft Excel worksheet prepared before the full-text review. The following information was extracted independently: author and publication details, study design and characteristics, participant demographics, intervention protocols, and outcome assessments. Accuracy was verified by a third reviewer [Y.W.Y], and disagreements were resolved through consultation with a fourth independent researcher [X.L].

For studies with missing numerical data or outcomes presented only in graphical format, corresponding authors were contacted for clarification. If no response was received, quantitative data were extracted from graphs using WebPlotDigitizer (version 4.1, Ankit Rohatgi, Belmont, CA, USA). In all data extraction processes, an intention-to-treat (ITT) approach was employed, meaning that all participants originally assigned to each treatment group were included, regardless of whether they completed the protocol or adhered to the treatment plan. Studies for which data remained unavailable were excluded from the meta-analysis.

### 2.5. Data Process

For the quantitative analysis, the mean, standard deviation (SD), and sample size were extracted for each group at both pre- and post-intervention timepoints. These values were used to calculate the mean change from baseline and its corresponding standard deviation for subsequent pooling of effect sizes.

The ${\mathrm{MD}}_{\mathrm{diff}}\;$, defined as the difference between the post-intervention mean and the pre-intervention mean, was calculated as follows:

(1) $${\mathrm{MD}}_{\mathrm{diff}}=M_{\mathrm{post}}-\;M_{\mathrm{pre}}$$

where ${\mathrm{MD}}_{\mathrm{diff}}\;$ is the raw mean difference, $M_{\mathrm{post}}$ is the reported mean post-intervention, and $M_{\mathrm{pre}}$ is the reported mean pre-intervention.

When only confidence intervals were reported, standard deviations were calculated using the following formula:

(2) $$\mathrm{SD}\;=\;\sqrt{N}\;\frac{{\mathrm{CI}}_{\mathrm{high}}-{\mathrm{CI}}_{\mathrm{low}}}{2t}$$

where SD is the standard deviation, N is the group sample size, ${\mathrm{CI}}_{\mathrm{high}}$ is the upper limit of the confidence interval, ${\mathrm{CI}}_{\mathrm{low}}$ is the lower limit of the confidence interval, and t is the t distribution with N − 1 degrees of freedom the respective confidence level.

The ${\mathrm{SD}}_{\mathrm{diff}}$ was calculated using a formula provided in the Cochrane Handbook [29]:

(3) $${\mathrm{SD}}_{\mathrm{diff}}\;=\;\sqrt{{{\mathrm{SD}}_{\mathrm{pre}}}^{2}\;+\;{{\mathrm{SD}}_{\mathrm{post}}}^{2}-2r\;\times \;{\mathrm{SD}}_{\mathrm{pre}}\;\times \;{\mathrm{SD}}_{\mathrm{post}}}$$

where ${\mathrm{SD}}_{\mathrm{diff}}$ is the standard deviation of the difference in means, ${\mathrm{SD}}_{\mathrm{pre}}$ is the standard deviation from pre-intervention, and ${\mathrm{SD}}_{\mathrm{post}}$ is the standard deviation from post-intervention.

As the original studies did not report Pearson’s correlation coefficients (r) for pre- and post-intervention outcomes, we assumed r = 0.5 based on recommendations from the Cochrane Handbook [30]. Sensitivity analyses where r was varied between 0.3 and 0.7 confirmed that the primary pooled estimates were robust.

(4) $$r=\frac{{{\mathrm{SD}}_{\mathrm{pre}}}^{2}+{{\mathrm{SD}}_{\mathrm{post}}}^{2}-{{\mathrm{SD}}_{\mathrm{change}}}^{2}}{2\;\times \;{\mathrm{SD}}_{\mathrm{pre}}\;\times \;{\mathrm{SD}}_{\mathrm{post}}}$$

### 2.6. Risk of Bias and Quality of Methods Assessment

Risk of bias was independently assessed by two reviewers [Y.W.H, Y.W.Y] using the Cochrane Risk of Bias 2 (RoB 2) tool, which evaluates bias across domains including random sequence generation, allocation concealment, blinding of participants and personnel, blinding of outcome assessment, incomplete outcome data, selective reporting, and other potential sources of bias. Discrepancies were resolved through discussion, with unresolved cases adjudicated by a third reviewer [Z.Z]. For non-randomized studies, bias was assessed using the Risk of Bias in Non-Randomized Studies of Interventions (ROBINS-I) tool, covering seven domains: confounding, participant selection, intervention classification, deviations from intended interventions, missing data, outcome measurement, and selection of reported results.

Methodological quality was additionally evaluated using the Physiotherapy Evidence Database (PEDro) scale (0–10 points), with scores ≥ 6 indicating high quality, 4–5 moderate quality, and ≤3 low quality [31].

### 2.7. Statistical Analysis

All statistical analyses were conducted in R (version 4.5.0, R Foundation for Statistical Computing, Vienna, Austria) [32].

#### 2.7.1. Overall Analytical Model

To account for the fact that several included studies reported multiple experimental groups or outcomes, a conventional two-level meta-analysis could violate independence assumptions and overestimate precision [33]. Therefore, a three-level random-effects model was employed [34], which partitions variance into sampling error (Level 1), within-study variance (Level 2), and between-study variance (Level 3), thereby accounting for effect-size dependency and hierarchical data structures [35].

The model’s robustness was further enhanced using cluster-robust variance estimation (CRVE) with small-sample corrections (specifically, the CR2 adjustment proposed by Bell and McCaffrey [36]) to handle correlated outcomes [37]. This adjustment provides more robust standard errors and wider, more conservative confidence intervals, which is crucial when the number of included studies is small [38]. All available effect sizes were retained rather than averaged or discarded, improving statistical power and precision [34]. Model parameters were estimated by restricted maximum likelihood (REML) and cross-validated with maximum likelihood (ML) estimation for robustness [39].

#### 2.7.2. Effect Size and Heterogeneity Assessment

The effect size was the standardized mean difference (SMD), calculated as Hedges’ g to correct for potential bias in small samples. Effect sizes were interpreted as trivial (<0.2), small (0.2–0.5), medium (0.5–0.8), or large (>0.8) [40]. Between-study heterogeneity was assessed using Cochrane’s Q, I^2^, τ^2^, and τ, with 95% confidence intervals (CI) and prediction intervals (PI) to quantify dispersion [41]. I^2^ served as the primary heterogeneity index and was interpreted as low (0–25%), moderate (25–50%), substantial (50–75%), or considerable (75–100%) [30]. Lastly, the statistical power for pooled estimates was evaluated with the “metameta” package [42].

#### 2.7.3. Moderator and Sensitivity Analyses

To explore potential sources of heterogeneity (when I^2^ > 25%), subgroup analyses and meta-regressions were performed [43]. Analyses focused on participant characteristics, intervention characteristics, and training protocols. The six subgroup variables were selected based on our pre-specified study protocol, theoretical considerations, and data availability: (1) menopause stage, (2) Tai Chi type, (3) body part, (4) intervention duration (weeks), (5) training frequency, and (6) intervention period. Subgroup analyses were conducted only when overall heterogeneity exceeded I^2^ > 25%.

Sensitivity analyses were conducted using leave-one-out methods to detect influential studies. Publication bias was examined with contour-enhanced funnel plots and Egger’s regression test, with *p* > 0.05 indicating no significant bias [44].

### 2.8. Certainty of the Evidence

The effectiveness evidence from each study was combined with quality scores for analysis. The Grading of Recommendations Assessment, Development, and Evaluation (GRADE) methodology was used to assess the certainty of evidence, categorizing it as “high,” “moderate,” “low,” or “very low” [45]. Two researchers independently conducted the GRADE assessment, resolving discrepancies through consensus. The evaluation criteria were as follows: (1) risk of bias: downgraded by one level for “some concerns” and two levels for “high risk” [46]; (2) inconsistency: downgraded by one level for moderate statistical heterogeneity (I^2^ > 25%) and two levels for high heterogeneity (I^2^ > 75%) [47]; (3) imprecision: downgraded by one level if statistical power was <80% or if effect directions were unclear [48]; (4) risk of publication bias: downgrade one level if Egger’s test < 0.05 [49].

## 3. Results

### 3.1. Studies Retrieved

A literature search was conducted across nine databases: PubMed, Web of Science, the Cochrane Library, MEDLINE (via EBSCO), SPORTDiscus (via EBSCO), Embase, and three Chinese databases: CNKI, VIP, and Wanfang Data. The initial search, performed on 1 June 2025, identified 373 publications. After removing 164 duplicates, 209 records were screened based on titles and abstracts. Additionally, 5 studies were identified through other sources.

**Figure 1:**
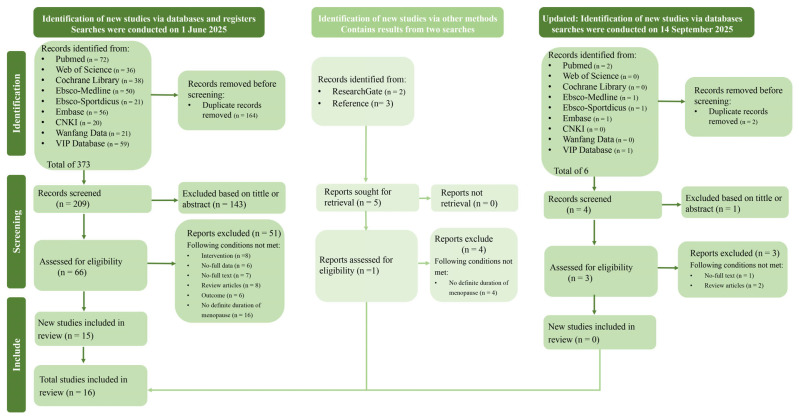
PRISMA flow diagram of study selection process.

After a full-text assessment, a total of 16 studies were deemed eligible and included in the final meta-analysis [15,16,17,50,51,52,53,54,55,56,57,58,59,60,61,62]. The detailed study selection process is shown in Figure 1.

### 3.2. Characteristics of Included Studies

**Figure 2:**
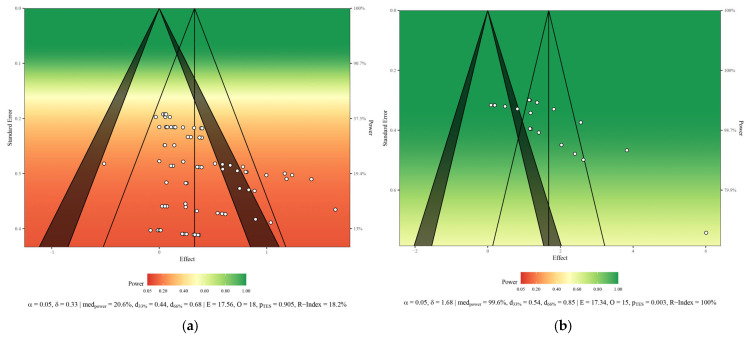

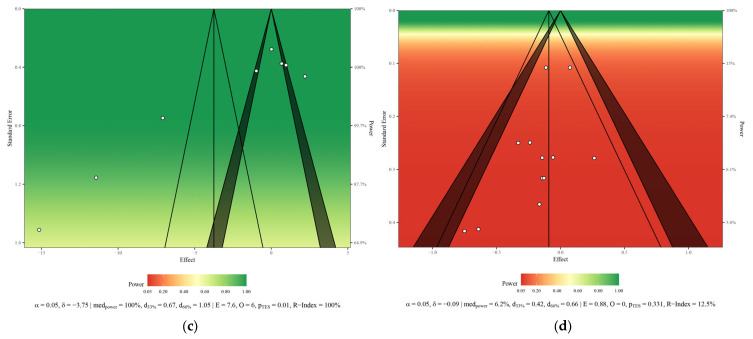
Sunset statistical power charts: (**a**) shows the plot for bone mineral density; (**b**) shows the plot for bone mineral metabolism; (**c**) shows the plot for bone mineral content; (**d**) shows the plot for bone turnover markers.

**Figure 3:**
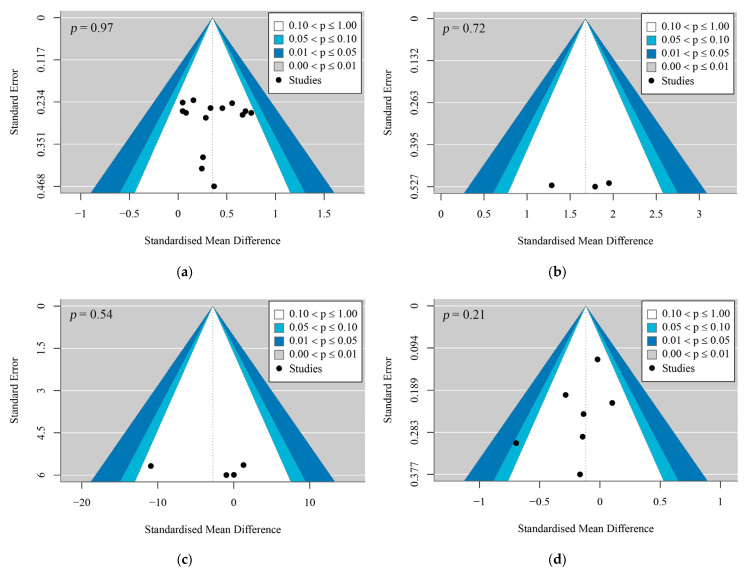
Funnel plots: (**a**) is the funnel plot for bone mineral density; (**b**) is the funnel plot for bone mineral content; (**c**) is the funnel plot for bone mineral metabolism; (**d**) is the funnel plot for bone turnover markers.

Among the 16 included studies, 15 were randomized controlled trials (RCTs) and 1 was non-randomized controlled trials. The total sample size was 1091, with individual study group sizes ranging from 22 to 344 participants, aged 49 to 64 years. Among them, 245 were in the perimenopausal stage (22.46%) and 846 in the postmenopausal stage (77.54%). Most studies provided detailed information on participant characteristics, study design, interventions, and follow-up procedures. A summary of baseline characteristics is provided in Appendix C. Statistical power diagrams and publication bias funnel plots for the combined outcomes are shown in Figure 2a–d and Figure 3a–d.

### 3.3. Primary Meta-Analysis Results

#### 3.3.1. Bone Mineral Density

A three-level meta-analysis was conducted to examine the effect of Tai Chi on BMD in menopausal women. Using restricted maximum likelihood (REML), the analysis included 78 effect sizes from 14 studies and found a significant positive impact of Tai Chi (k = 78, SMD = 0.31, 95% CI [0.16, 0.45], I^2^-2 = 0%, I^2^-3 = 36.62%, PI [−0.11, 0.82], *p* < 0.01), compared to the CON group. Please refer to Appendix D for the detailed forest plot.

#### 3.3.2. Bone Mineral Content

To evaluate the effect of Tai Chi on BMC in menopausal women, this study conducted a three-level meta-analysis using REML estimation. Using REML estimation, the analysis included 18 effect sizes from 3 studies. Tai Chi significantly improved BMC (k = 18, SMD = 1.63, 95% CI [0.86, 2.40], I^2^-2 = 0%, I^2^-3 = 91.46%, PI [−1.28, 4.54], *p* < 0.01), compared to the CON group. Due to high heterogeneity (I^2^ = 91.46%), the CR2 adjustment was applied to strengthen the effect size estimate. After adjustment, the effect remained significant (SMD = 1.63, 95% CI [0.64, 2.62], *p* = 0.02), confirming a consistent positive impact of Tai Chi on BMC even after accounting for potential bias.

#### 3.3.3. Bone Mineral Metabolism

A three-level multivariate meta-analysis using REML was performed to evaluate the effects of Tai Chi on BMM in menopausal women. The model synthesized 8 effect sizes from 4 independent studies. The pooled effect was not statistically significant (k = 8, SMD = −2.71, 95% CI [−9.54, 4.11], I^2^-2 = 79.54%, I^2^-3 = 18.74%, PI [−18.34, 12.91], *p* = 0.38).

Given the small number of studies and substantial heterogeneity (I^2^-total = 98.28%), a small-sample correction was applied using the CR2 robust variance estimator. The adjusted effect size remained negative and non-significant (SMD = −2.71, 95% CI [−12.00, 6.59], *p* = 0.421), corroborating the primary analysis. Collectively, these results suggest no consistent impact of Tai Chi on BMM in this population, with the wide confidence intervals precluding any meaningful inference from this analysis.

#### 3.3.4. Bone Turnover Markers

The effects of Tai Chi on BTMs in menopausal women were examined through a three-level multivariate meta-analysis with REML. examine the overall effect of Tai Chi on BTMs in menopausal women. The analysis synthesized 12 effect sizes from 7 independent studies. The pooled effect was not statistically significant (k = 12, SMD = −0.10, 95% CI [−0.27, 0.08], I^2^-2 = 0%, I^2^-3 = 58.71%, PI [−0.34, 0.14], *p* = 0.25), indicating no consistent impact of Tai Chi on BTMs.

Given the considerable between-study heterogeneity (I^2^-total = 58.71%), we applied the CR2 robust variance estimator to improve small-sample inference. After adjustment, the effect remained negative and non-significant (SMD = −0.10, 95% CI [−0.35, 0.16], *p* = 0.29), reinforcing the primary finding. The lack of a significant effect could be due to insufficient statistical power, given the moderate sample size.

### 3.4. Secondary Meta-Analysis Results

#### 3.4.1. Bone Mineral Density

The primary analysis indicated that Tai Chi exercise significantly improved BMD (SMD = 0.31, *p* < 0.01), with moderate to low heterogeneity (I^2^-total = 36.62%). To better understand the impact of Tai Chi on BMD, this study explored the heterogeneity of its effects from multiple dimensions. Specifically, the effects of Tai Chi on BMD were examined with a focus on different menopausal stages, body regions, and types of Tai Chi exercise. The detailed subgroup analysis results are shown in Table 2.

In the subgroup analysis by menopausal stage, a significant moderating effect was observed (*p* < 0.01, QE = 96.46). In perimenopausal women, the pooled effect size was significant (SMD = 0.53, 95% CI [0.35, 0.71], *p* < 0.001, I^2^-2 = 0%, I^2^-3 = 49.62%). In postmenopausal women, the pooled effect was smaller (SMD = 0.22, 95% CI [0.06, 0.37], *p* = 0.006, I^2^-2 = 30.16%, I^2^-3 = 0%). A pairwise comparison indicated that the difference between groups was significant (ΔSMD = −0.32, *p* < 0.01).

The subgroup analysis by body part revealed significant between-site differences (*p* = 0.03), indicating that treatment effects varied by body parts. Residual heterogeneity within the subgroups remained significant (QE = 102.42, *p* < 0.01). Significant improvements were observed at several body parts, including the lumbar spine L2–L4 (SMD = 0.47, 95% CI [0.28, 0.65], *p* < 0.001, I^2^ = 51.99%), femoral neck (SMD = 0.37, 95% CI [0.14, 0.59], *p* < 0.01, I^2^ = 72.44%), greater trochanter (SMD = 0.33, 95% CI [0.10, 0.56], *p* = 0.005, I^2^ = 41.19%), thigh (SMD = 0.32, 95% CI [0.04, 0.60], *p* = 0.03, I^2^ = 9.83%), Ward’s triangle (SMD = 0.27, 95% CI [0.03, 0.51], *p* = 0.029, I^2^ = 21.75%), upper limb (SMD = 0.29, 95% CI [0.03, 0.56], *p* = 0.030, I^2^ = 3.64%), and trunk (SMD = 0.96, 95% CI [0.42, 1.49], *p* < 0.001, I^2^ = 0). In contrast, no significant effects were detected at the calcaneus (SMD = 0.05, *p* = 0.75), tibia (SMD = 0.06, *p* = 0.72), pelvis (SMD = 0.14, *p* = 0.45), or whole body (SMD = 0.03, *p* = 0.86).

In the subgroup analysis by Tai Chi type, heterogeneity was significant (QE = 110.56, *p* < 0.01). Tai Chi Quan showed significant benefits (SMD = 0.27, 95% CI [0.11, 0.43], *p* = 0.001, I^2^ = 37.52%), as did Tai Chi Rouli Ball (SMD = 0.60, 95% CI [0.33, 0.87], *p* < 0.001, I^2^ = 63.33%), whereas Tai Chi Push Hands did not show a significant effect (SMD = 0.33, 95% CI [−0.08, 0.74], *p* = 0.12, I^2^ = 0%). The overall moderating effect of exercise type was not statistically significant (*p* = 0.12), indicating that no significant between-group differences were observed among Tai Chi forms. Nevertheless, pairwise comparison indicated Tai Chi Rouli Ball was significantly more effective than Tai Chi Quan (ΔSMD = 0.33, 95% CI [0.02, 0.64], *p* = 0.04). However, given the marginal significance and the small number of studies included for Rouli Ball, this should be considered a preliminary finding.

**Figure 4:**
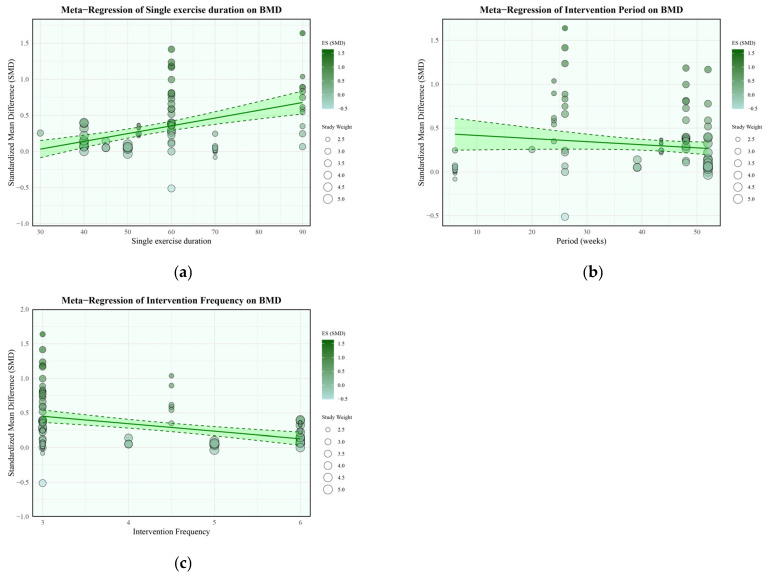
Meta-Regression plots: (**a**) is the Meta-Regression plot for single exercise duration (minutes) in bone mineral density; (**b**) is the Meta-Regression plot for intervention period in bone mineral density; (**c**) is the Meta-Regression plot for intervention frequency in bone mineral density.

A meta-regression was conducted to identify if key exercise characteristics influenced BMD improvements (Figure 4). The analysis revealed that exercise duration (e.g., minutes per session) was a significant positive predictor of the effect (β = 0.0108, 95% CI [0.006, 0.0149], *p* < 0.01). This indicates that for each additional minute of exercise duration per session, the effect on BMD was associated with a 0.0108 standard deviation increase. In contrast, the total intervention period (e.g., total weeks) did not significantly influence the effect (β = −0.0035, 95% CI [−0.0081, 0.0011], *p* = 0.13). Interestingly, practice frequency (e.g., times per week) was a significant negative predictor (β = −0.1080, 95% CI [−0.1539, −0.0622], *p* < 0.01). This suggests that for each additional practice session per week, the effect on BMD was associated with a 0.1080 standard deviation decrease. As this finding is counterintuitive, it will be explored further in the Section 4.

In addition to the individual moderators, we further evaluated potential interaction effects. A three-level meta-regression model revealed a significant interaction between menopausal stage and body parts (QM = 33.99, *p* < 0.01). The detailed predicted effect sizes for each combination of stage and body part are presented in Table 3. Overall, the analysis indicated that the effects of Tai Chi were generally stronger for perimenopausal women (e.g., Lumbar spine L2–L4: SMD = 0.81) compared to postmenopausal women (e.g., Lumbar spine L2–L4: SMD = 0.25) at key skeletal sites. The interaction forest plot for this analysis is available in the Appendix E.

Overall, subgroup and meta-regression analyses indicated that the effects of Tai Chi on BMD were moderated by menopausal stage, skeletal sites, and exercise type, with perimenopausal women, trunk and spine regions, and Tai Chi Rouli Ball showing greater benefits; moreover, session duration and practice frequency emerged as significant predictors, while an interaction between menopausal stage and body sites further highlighted site-specific effects.

#### 3.4.2. Bone Mineral Content

As shown in Table 4, the residual heterogeneity was significant (QE = 108.38, *p* < 0.01) in the body site subgroup analysis. Significant effects were observed at the hip (SMD = 2.22, 95% CI [0.24, 4.20], *p* = 0.03, I^2^ = 96.57%), lumbar spine L2–L4 (SMD = 1.51, 95% CI [0.01, 3.02], *p* < 0.05, I^2^ = 0%), and upper limb (SMD = 2.60, 95% CI [0.19, 5.00], *p* = 0.04, I^2^ = 0%). In contrast, no significant effect was observed at the pelvis (SMD = 1.38, *p* = 0.23, I^2^ = 0%) or thigh (SMD = 1.15, *p* = 0.31, I^2^ = 0%). Pairwise comparisons between body sites were nonsignificant (*p* = 0.58). In the subgroup analysis by exercise duration, the residual heterogeneity was significant (QE = 122.60, *p* < 0.01). The 60 min subgroup showed a significant effect (SMD = 1.95, 95% CI [0.84, 3.07], *p* < 0.01, I^2^-2 = 0%, I^2^-3 = 87.81%), and the 90 min subgroup also showed a significant effect (SMD = 1.54, 95% CI [0.74, 2.34], *p* = 0.001, I^2^-2 = 0%, I^2^-3 = 91.08%). The between-group difference was not significant (ΔSMD = 2.36, *p* = 0.53). In the subgroup analysis by exercise frequency, the residual heterogeneity was significant (QE = 129.61, df = 16, *p* < 0.01). Significant effects were observed for <4 sessions/week (SMD = 1.63, 95% CI [0.82, 2.43], *p* < 0.01, I^2^-2 = 0%, I^2^-3 = 93.85%) and >4 sessions/week (SMD = 1.79, 95% CI [0.64, 2.94], *p* < 0.01, I^2^-2 = 0%, I^2^-3 = 50.63%). The between-group difference was nonsignificant (ΔSMD = 0.16, *p* = 0.81).

Taken together, subgroup analyses by body site, exercise duration, and frequency all showed significant effects on BMC, with exercise frequency demonstrating relatively lower between-study heterogeneity.

#### 3.4.3. Bone Mineral Metabolism

Although the overall effect of BMM did not reach statistical significance, substantial heterogeneity was observed (I^2^-total = 98.28%). Therefore, subgroup analyses were conducted to explore potential sources of heterogeneity. The detailed subgroup analysis results are shown in Table 5.

Specifically, we conducted subgroup analyses based on menopausal stage (perimenopausal vs. postmenopausal), exercise duration (60 min vs. 90 min), and exercise frequency (<4 vs. >4 times/week). However, none of these variables revealed significant subgroup differences (all *p* > 0.05). Furthermore, substantial heterogeneity persisted within most subgroups (I^2^ > 75%), suggesting that these factors did not sufficiently explain the observed variance.

These findings highlight the necessity of future high-quality studies to better identify the determinants of variability in BMM outcomes.

#### 3.4.4. Bone Turnover Markers

Although the overall effect of BTMs did not reach statistical significance, substantial heterogeneity was observed (I^2^-total = 58.71%), prompting further subgroup analyses to explore potential sources of heterogeneity. The detailed subgroup analysis results are shown in Table 6.

Analyses by menopausal stage, exercise duration, and weekly training frequency did not reveal any significant differences between groups (all *p* > 0.05).

However, a subgroup analysis by intervention period revealed an interesting trend-level finding that warrants future research: short-term interventions (<1 year) showed a small but statistically significant reduction in BTMs (SMD = −0.27, 95% CI [−0.52, −0.03], *p* = 0.03, I^2^ = 0%), whereas long-term interventions (≥1 year) showed no effect (SMD = −0.01, *p* = 0.96).

### 3.5. Sensitivity Analysis

To evaluate the robustness of the pooled effect estimates, sensitivity analyses were performed using a leave-one-out approach, where each study cluster was sequentially removed to assess its influence on the overall effect size and statistical significance. For the BMD, BMC, BMM and BTMs outcomes, the leave-one-out results consistently demonstrated that the pooled estimates were highly robust. Excluding any single study did not alter the direction or statistical significance of the overall effects.

Specifically, the overall effect sizes for BMD and BMC were significant, while BMM and BTMs showed non-significant effects. To quantify this stability, the maximum observed change in the pooled SMD was 0.016 for BMD, 0.09 for BMC, 1.73 for BMM, and 0.0586 for BTMs. While the maximum change for BMM was numerically larger, it was statistically negligible given the outcome’s highly non-significant result (*p* = 0.42) and very wide confidence interval. Thus, no single study cluster significantly influenced the overall results. For detailed forest plots and sensitivity statistics for each outcome category, please refer to Appendix F.

### 3.6. Risk of Bias and Methodological Quality

**Figure 5:**
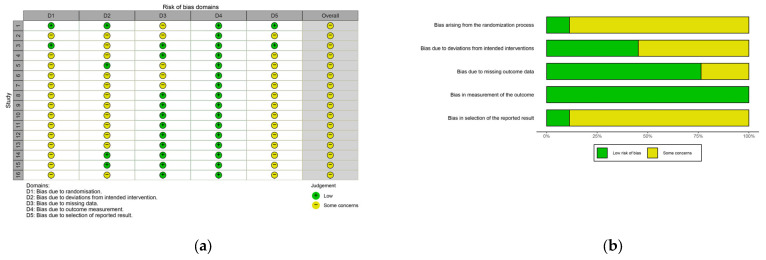
Risk of bias assessment diagram. Study 1 is Xin et al., 2024 [50]; Study 2 is Qin et al., 2002 [51]; Study 3 is Wayne et al., 2012 [52]; Study 4 is Xiao et al., 2015 [16]; Study 5 is Cheng et al., 2022 [15]; Study 6 is Chan et al., 2004 [17]; Study 7 is Zhou et al., 2003 [53]; Study 8 is Zhao et al., 2020 [54]; Study 9 is Zhou et al., 2005 [55]; Study 10 is Du et al., 2014 [56]; Study 11 is Liu et al., 2021 [57]; Study 12 is Guo et al., 2014 [58]; Study 13 is Mao et al., 2009 [59]; Study 14 is Xue et al., 2015 [60]; Study 15 is Xu et al., 2017 [61]; Study 16 is Zhou et al., 2004 [62]. (**a**) Risk of bias summary plot (**b**) Risk of bias traffic light plot.

Using the RoB 2.0 tool, several methodological concerns were identified across included studies. “Some concerns” were noted in 87% of studies for the randomization process (D1) and selective reporting (D5), largely due to unclear allocation concealment and lack of pre-registration. In Domain 2, 75% of studies lacked blinding, and 31% showed issues with missing outcome data (D3). Notably, all studies were rated low risk in outcome measurement (D4). These limitations could introduce a bias towards overestimating treatment effects. However, since the findings for BMM and BTMs were consistently null, this bias is unlikely to be the sole explanation for the observed heterogeneity. These findings suggest that while methodological limitations exist in certain domains, the overall evidence remains informative and should be interpreted with appropriate caution. The detailed results were demonstrated in Figure 5a,b.

The methodological quality of the included studies was evaluated using the Physiotherapy Evidence Database (PEDro) scale, as shown in Table 7. The average PEDro score across all studies was 6.31, indicating a high overall methodological quality. Specifically, 87% of the studies were rated as high quality (score ≥ 6), 13% as moderate quality (scores between 4 and 5), and none of the studies were considered low quality (score < 3).

### 3.7. Results of the Certainty of the Evidence

Overall, the certainty of the evidence ranged from low to moderate across the assessed outcomes, as shown in Table 8. Moderate certainty was found for the effect on BMD. In contrast, the certainty of evidence for the effects on BMC, BMM and BTMs was low, due to the high heterogeneity and imprecision.

## 4. Discussion

This three-level meta-analysis systematically evaluated the effects of Tai Chi exercise on bone health in menopausal women. The findings demonstrated that Tai Chi exercise significantly improved BMD and BMC, with greater benefits observed in perimenopausal women, specific skeletal sites (e.g., lumbar spine and femoral neck), and certain forms of Tai Chi (e.g., Tai Chi Rouli Ball). In contrast, Tai Chi exercise showed no consistent significant effects on BMM or BTMs, suggesting that its benefits are primarily related to enhancing bone mass rather than metabolic regulation. Moreover, exercise duration and frequency emerged as significant predictors of BMD improvement, while short-term interventions and higher weekly frequency showed trend-level effects on BTMs. Overall, Tai Chi exercise appears to exert positive effects on bone health in menopausal women, although further high-quality studies are needed to clarify its mechanisms and long-term efficacy.

### 4.1. Bone Mineral Density

The three-level meta-analysis indicated that Tai Chi exercise could modestly but clinically meaningfully improve BMD in menopausal women (SMD = 0.31). However, the overall effect remained limited, with both menopausal stage and site-specific variations. Perimenopausal women showed more pronounced improvements at weight-bearing sites such as the lumbar spine L2–L4 (SMD = 0.47), femoral neck (SMD = 0.37), and greater trochanter (SMD = 0.33), whereas the effect in postmenopausal women was relatively weaker (SMD = 0.22). Subgroup analysis by exercise modality further revealed that Tai Chi Rouli Ball (SMD = 0.60) outperformed traditional Tai Chi Quan (SMD = 0.27), suggesting that training mode can influence skeletal adaptation. However, for Tai Chi Push Hands, no statistically significant effect was observed (SMD = 0.33, *p* > 0.05). This lack of statistical significance is most likely attributable to the low number of included effect size in this subgroup (k = 5), resulting in insufficient statistical power to detect a true effect. Therefore, findings for Tai Chi Push Hands should be interpreted as exploratory and not conclusive regarding its ineffectiveness.

While the effect size for postmenopausal women was modest (SMD = 0.22), its clinical relevance should be interpreted from a broader public health perspective. According to the landmark meta-analysis by Marshall et al., the general relative risk for fractures is 1.5 (95% CI: 1.4–1.6) for each standard deviation drop in BMD. More importantly, the authors highlighted a critical exception: for hip fractures predicted by hip BMD, the relative risk surges to 2.6 (95% CI: 2.0–3.5), a figure that underscores the unique vulnerability of this site [63]. In this high-risk context, the mean increase of 0.22 standard deviations achieved through a safe and low-cost intervention like Tai Chi represents a clinically relevant contribution to fracture risk reduction. Perhaps more critically, the primary value of Tai Chi in preventing osteoporotic fractures extends beyond its direct influence on BMD. A major pathway to fracture in this population is falling. Tai Chi is a multicomponent therapy renowned for its substantial benefits in improving balance and neuromuscular control. In fact, a recent meta-analysis of 24 randomized controlled trials demonstrated that Tai Chi practice can reduce the risk of falls in older adults by a significant 24% (RR = 0.76, 95% CI: 0.71 to 0.82) [64]. Consequently, the overall clinical utility of Tai Chi may be substantially attributable to the combination of a modest, direct effect on bone preservation and a larger, more critical effect on fall prevention.

Importantly, our moderator analyses also provided further insights. Subgroup analyses revealed a statistically significant moderating effect of ‘Body Part’ (Pb = 0.0273), suggesting that Tai Chi’s effects on bone health vary significantly across different body sites. This variation likely reflects differential biomechanical responses, with weight-bearing sites often exhibiting greater sensitivity to mechanical loading. In contrast, the ‘Tai Chi Type’ moderator did not show a statistically significant between-subgroup difference (Pb = 0.1192), indicating that the Tai Chi style practiced (e.g., Tai Chi Quan vs. Tai Chi Push Hands) did not significantly moderate the overall effect on bone health. This non-significant finding may reflect either (a) genuinely similar osteogenic stimuli provided by the low-to-moderate intensity across different Tai Chi styles, or (b) insufficient statistical power to detect a true moderating effect, particularly given the small number of studies in the Tai Chi Push Hands subgroup (k = 5). Furthermore, an additional interaction meta-regression analysis demonstrated a significant moderating effect of menopausal stage × skeletal site (QM = 33.9981, *p* = 0.0054). Predicted effect sizes were particularly strong in perimenopausal women at the femoral neck (SMD = 0.50), greater trochanter (SMD = 0.61), and lumbar spine L2–L4 (SMD = 0.81). These findings indicate substantial benefits at critical skeletal locations. In contrast, postmenopausal women exhibited smaller improvements limited to the femoral neck (SMD = 0.28) and lumbar spine (SMD = 0.25), with no significant effect at the greater trochanter or upper limb. These findings underscore that Tai Chi’s skeletal benefits are most evident during the perimenopausal transition, whereas postmenopausal women display reduced responsiveness, likely due to estrogen deficiency and progressive deterioration of trabecular microarchitecture [65].

The underlying mechanisms may operate at multiple levels. First, Tai Chi’s slow weight-shifting, semi-squatting, and single-leg support movements provide sustained, multidirectional mechanical loading to the lumbar spine and hip, consistent with the mechanostat theory [66]. Enhanced lower-limb and trunk muscle strength further contributes to skeletal loading through the muscle–bone unit [67] and to the improvements in balance and stability that underpin the fall prevention benefits discussed earlier. Second, mechanical signals induced by exercise may activate the Wnt/β-catenin pathway, suppress sclerostin expression, and stimulate osteoblast activity [68,69], while regular practice may also attenuate inflammatory cytokines and oxidative stress, thereby mitigating bone loss [70,71]. Third, residual estrogen in perimenopausal women may amplify bone sensitivity to mechanical stimuli, helping to explain the stage-specific differences observed in this study [72,73]. The superior effect of Tai Chi Rouli Ball may be attributed to larger movement amplitude, additional resistance from the implement, and improved exercise adherence [65,74].

Meta-regression analyses yielded additional insights regarding exercise dosage. Longer exercise duration per session significantly predicted greater BMD improvement, whereas intervention length showed no significant effect. Practice frequency, unexpectedly, showed a negative association with BMD improvement. This paradoxical finding requires cautious interpretation, as it may reflect interacting physiological and methodological factors rather than a straightforward causal relationship.

The negative association observed between intervention frequency and BMD is counterintuitive. One possible explanation could be an overtraining effect. While the included interventions were of moderate intensity, bone remodeling is a process that takes several weeks, and training that is too frequent without adequate recovery could potentially disrupt this cycle [75]. However, this interpretation is highly speculative. This finding is limited by several factors. First, we have no direct measurements of fatigue, recovery, or bone turnover markers from the included studies. Second, a meta-regression analysis can only identify a correlation and cannot prove causation. It is also possible that this finding is not a true biological effect but is due to confounding by other unmeasured variables (e.g., specific exercise content, participant adherence, or dietary factors) that may be associated with the higher-frequency protocols. Therefore, this result should be interpreted with extreme caution, and further primary research is needed to explore this hypothesis.

Several methodological limitations warrant acknowledgment, which temper the interpretation of our findings on exercise frequency. First, the quality of some included studies raised concerns regarding potential confounding bias. Second, our analysis was inherently constrained by the available data, as the minimum intervention frequency was three sessions per week, precluding any assessment of lower-frequency protocols. Despite these constraints, we observed a potential negative association trend between frequencies of 3–6 sessions/week. One speculative interpretation is that three sessions per week may approximate an optimal frequency, an idea supported by other reviews which have noted thrice-weekly as a prevalent frequency for promoting bone health [76]. However, we must emphasize that this remains highly tentative. Given the methodological limitations of the included studies and the absence of low-frequency data, we cannot distinguish a true dose–response effect from statistical artifacts or publication bias. Therefore, to resolve this uncertainty, rigorously designed dose–response studies are essential. Future research should systematically compare different frequencies, including 1–2 sessions/week, to definitively characterize the optimal protocol for this population.

In summary, Tai Chi appears particularly effective in improving bone mass at the lumbar spine and hip among perimenopausal women, supporting its role as a preventive strategy against early menopausal bone loss. Subgroup and meta-regression analyses indicate that the observed heterogeneity is largely explained by menopausal stage and skeletal site. As a safe and low-impact modality, Tai Chi may therefore be recommended as an adjunctive approach to osteoporosis prevention in this population.

### 4.2. Bone Mineral Content

The present three-level meta-analysis showed that Tai Chi exercise significantly improved BMC in menopausal women, with a large effect size (SMD = 1.63). This effect exceeded that observed for BMD, suggesting that BMC may be a more sensitive marker of skeletal adaptation to Tai Chi. The result remained robust under CR2 variance adjustment, despite the limited number of studies. However, the high between-study heterogeneity (I^2^ > 90%) indicates substantial variability across trials, warranting a deeper investigation into its potential sources.

Our subgroup analyses provided some initial insights, suggesting that skeletal site and higher exercise frequency (>4 sessions/week) could partially explain this variance. However, significant residual heterogeneity still remained for BMC, likely reflecting issues common to our other analyses. This variance probably stems from several key, unmeasured sources.

First, the “Tai Chi” intervention itself is heterogeneous, including distinct modalities like traditional Tai Chi Quan, Push hands, and Tai Chi Rouli Ball, each with unique biomechanical demands [77,78]. Moreover, this is further confounded by the lack of specification of Tai Chi styles (e.g., Yang, Sun) in many trials.

Second, a crucial source of heterogeneity is the pooling of physiologically distinct populations under the single “perimenopausal” label. This term overlooks the dynamic nature of the transition, which the STRAW + 10 criteria [79] formally delineate into an early phase (Stage-2, variable/high estrogen) and a late phase (Stage-1, low estrogen) [80]. Lumping these groups, who inherently differ in baseline bone turnover and skeletal sensitivity, creates significant unmeasured intra-group variance.

Lastly, methodological inconsistencies likely contributed. These include poorly reported adherence, which obscures the true exercise dose, and variations in measurement techniques, such as the use of different DXA densitometers across study sites [81]. The confluence of these factors provides a plausible, multi-faceted basis for the high degree of heterogeneity observed.

The mechanisms underlying these improvements may overlap with those observed for BMD but could be more closely linked to structural bone adaptations. Tai Chi involves slow, weight-bearing movements, semi-squats, and dynamic balance shifts, which generate mechanical loading at the hip and lumbar spine, stimulating bone modeling and mineral accretion [82]. According to the mechanostat theory, these stimuli promote osteogenesis by activating osteoblast activity and enhancing bone mineral deposition [83]. In addition, muscle strengthening induced by Tai Chi provides further loading through the muscle–bone unit, particularly in the hip and upper limb. From a molecular perspective, mechanical strain may activate Wnt/β-catenin signaling and downregulate sclerostin, promoting bone formation [84,85]. Furthermore, Tai Chi’s systemic effects—such as improving balance, cardiometabolic health, and reducing inflammation—may support both bone formation and mineral retention.

These findings suggest that Tai Chi has a significant effect on BMC in menopausal women, especially at clinically relevant skeletal sites. However, due to the small number of studies and high heterogeneity, the findings should be interpreted cautiously. Future research should prioritize larger, rigorously designed trials. Crucially, these trials need to adopt standardized reporting for Tai Chi protocols (style, intensity), participant characteristics (menopausal stage), intervention adherence, and outcome measurement techniques to validate these findings and allow for a more robust investigation into the sources of heterogeneity.

### 4.3. Bone Mineral Metabolism

The current meta-analysis showed that Tai Chi did not produce a statistically significant effect on BMM in menopausal women, with a negative but nonsignificant pooled effect size. Even after CR2 adjustment, the results remained null, and the wide confidence intervals underscored the uncertainty of available evidence. This contrasts with the positive effects observed for BMD and BMC, suggesting that Tai Chi’s skeletal benefits may primarily manifest at the structural level rather than the biochemical level.

As with the BMC findings, the high heterogeneity in BMM results likely stems from the mix of different intervention types, unstratified menopausal stages (per the STRAW + 10 framework), and inconsistent measurement techniques.

Mechanistically, a primary explanation for the null findings is that the osteogenic stimulus provided by Tai Chi may fall below the threshold required to elicit significant systemic changes in circulating bone mineral metabolism [86]. While Tai Chi’s slow, weight-bearing movements appear adequate to induce localized, structural adaptations in bone tissue (as reflected in BMD/BMC), they may lack the mechanical intensity needed to significantly alter these systemic biochemical indices [87]. Moreover, BMM are inherently variable, influenced by circadian rhythm, nutrition, vitamin D status, and menopausal hormone profile [88]. The short intervention durations (typically 12–24 weeks) in most trials further reduce the likelihood of capturing meaningful metabolic changes [89,90]. Additionally, estrogen deficiency in menopausal women may blunt the responsiveness of bone metabolism to low- to moderate-intensity exercise [87,91].

In sum, Tai Chi shows no consistent effects on BMM in menopausal women. The substantial heterogeneity across studies likely reflects methodological inconsistencies and biomarker variability rather than true physiological effects. Nevertheless, the contrast with positive findings for BMD and BMC suggests that Tai Chi’s skeletal benefits may be primarily structural. Future research should employ standardized biomarker panels and longer intervention periods. Crucially, to clarify any potential systemic effects, these trials must adopt the standardized reporting for intervention protocols, participant characteristics (including STRAW + 10 stages), and adherence that was detailed in our preceding discussion.

### 4.4. Bone Turnover Markers

This meta-analysis found that Tai Chi had no significant effect on BTMs in menopausal women, with a small and nonsignificant pooled effect size (SMD = −0.10). The null result persisted after CR2 adjustment, confirming that the lack of effect was robust. Unlike the positive effects observed for BMD and BMC, Tai Chi did not influence biochemical markers of bone turnover, suggesting a disconnect between structural adaptations and metabolic responses. This is consistent with previous research findings [76].

Subgroup analyses further reinforced this interpretation. No significant effects were observed when stratified by menopausal stage, exercise duration (<45 vs. ≥45 min), or training frequency (<4 vs. 4 vs. >4 sessions/week). However, a noteworthy pattern emerged in terms of intervention length: short-term interventions (<1 year) produced a small but statistically significant reduction in BTMs (SMD = −0.27, *p* = 0.031), whereas long-term interventions (≥1 year) showed no effect. Similarly, a trend-level reduction was observed in programs with higher weekly frequency (>4 sessions/week), though the effect did not reach statistical significance (SMD = −0.49, *p* = 0.070). These findings suggest that while Tai Chi may exert transient or intensity-dependent influences on bone metabolism, such effects are not sustained over time and may dissipate with prolonged practice.

Mechanistically, these results highlight a nuanced, time-dependent disconnect between structural and biochemical responses. The finding that interventions shorter than one year significantly reduced BTMs (SMD = −0.27) suggests a possible anti-resorptive effect during the initial phase of adaptation. In the high-turnover and uncoupled state of menopause, where bone resorption is pathologically elevated, the novel mechanical loading from Tai Chi may preferentially exert an anti-resorptive influence. This targeted suppression of excessive osteoclast activity leads to an overall decrease in bone turnover, a finding powerfully supported by a recent meta-analysis, which demonstrated that the effects of exercise on bone resorption markers (e.g., CTX) in postmenopausal women are most pronounced within the first 6 months of intervention, diminishing thereafter [92].

The lack of effect in long-term interventions (≥1 year) may reflect the establishment of a new homeostatic set-point. After this initial anti-resorptive phase, bone turnover likely returns to a new, more balanced equilibrium where structural gains are maintained without significant systemic metabolic shifts [93]. Moreover, the inherent variability of BTMs, influenced by numerous factors such as circadian rhythm and nutritional status, can obscure subtle exercise-induced changes [94,95]. In menopausal women specifically, this challenge is amplified, as estrogen deficiency is known to reduce the responsiveness of bone turnover to exercise [96].

In summary, Tai Chi does not appear to exert consistent long-term effects on BTMs in menopausal women. However, the observed short-term reductions and trends with higher training frequencies suggest potential windows of metabolic responsiveness. The certainty of these exploratory findings is nevertheless tempered by the substantial heterogeneity across trials. This challenge, as detailed in Section 4.2, likely reflects the profound methodological and clinical variance inherent in the primary literature, spanning from the intervention protocols to participant characteristics. Therefore, while the observed trends carry exploratory value, they must be interpreted with caution. Future studies should not only use standardized biomarkers and longer follow-ups but must also adopt the rigorous, standardized reporting for intervention details, participant staging (via STRAW + 10), and adherence that is necessary to truly clarify whether these transient effects are clinically meaningful.

### 4.5. Limitations

While this meta-analysis employed a rigorous three-level model, its findings must be interpreted in light of several limitations. The evidence base is constrained by the methodological quality of the primary studies, with many trials showing deficiencies in randomization, allocation concealment, and blinding of assessors. The unavoidable inability to blind participants and instructors introduces a risk of performance bias. Furthermore, insufficient reporting on adherence and missing data could affect the accuracy of the effect sizes. A key methodological limitation of this analysis is the imputation of the pre-post correlation coefficient (r = 0.5). Although standard practice for unreported data, any deviation of the true correlation from this assumed value could impact the precision of the pooled estimates by affecting the variance of the effect sizes.

Several other limitations warrant mention. First, as detailed in our discussion, significant clinical and methodological heterogeneity (e.g., diverse interventions, unstratified populations per STRAW + 10, varied control groups) was a major source of the observed variance. Second, the evidence base was limited for certain outcomes (BMC, BMM, BTMs), restricting statistical power and rendering these findings exploratory. Third, the relatively short duration of most interventions (<1 year) may have been insufficient to capture the full spectrum of skeletal adaptation, especially for slower-changing or transient systemic markers. To address these limitations, future research should prioritize large, long-term RCTs with standardized protocol reporting, rigorous adherence monitoring, and participant stratification using STRAW + 10 criteria.

## 5. Conclusions

This study employed a three-level meta-analytic approach to assess the impact of Tai Chi on bone health in menopausal women. The analysis demonstrated significant improvements in BMD and BMC, especially among perimenopausal women and at weight-bearing skeletal sites, such as the lumbar spine and femoral neck. However, Tai Chi did not consistently show significant effects on BMM or BTMs, indicating that its primary benefits are related to enhancing bone mass rather than influencing metabolic regulation. While longer session duration was a positive predictor of BMD improvements, the unexpected negative association with training frequency requires further investigation and should not be used to guide practice at this time. There are several limitations to consider. Some studies had small sample sizes and limited population diversity, which may affect the ability to generalize the findings. A significant challenge was the exceptionally high heterogeneity observed in certain outcomes, particularly BMC, which likely stemmed from multiple sources, including variations in Tai Chi protocols, unstratified menopausal stages, and methodological inconsistencies. Additionally, the inconsistent measurement of BMM and BTMs across studies makes it challenging to interpret the metabolic effects accurately. Despite these limitations, this meta-analysis provides compelling evidence for Tai Chi as a beneficial, accessible intervention for enhancing bone mass in menopausal women, underscoring the need for more standardized and rigorously reported studies in this field.

## Figures and Tables

**Table 1:** Studies eligibility criteria.

	Inclusion	Exclusion
P	Menopausal women, including perimenopausal (<12 months since last menstruation) and postmenopausal (≥12 months since last menstruation), with no restriction on age or occupation.	Non-menopausal women; men
I	Tai Chi exercise	Non–Tai Chi interventions
C	Usual physical activity was defined as maintaining habitual daily routines without structured exercise training, encompassing unavoidable low-intensity aerobic activities (e.g., walking, household chores).	Studies without control groups or with high-intensity exercise as control.
O	Bone health outcomes in any body part, including: ① Bone Mineral Density (BMD, assessed by DXA at lumbar spine, femoral neck, etc.) ② Bone Mineral Content (BMC, absolute mineral content) ③ Bone mineral metabolism (BMM, e.g., serum calcium, phosphate, vitamin D, PTH) ④ Bone Turnover Markers (BTMs, e.g., osteocalcin, CTX, P1NP)	No bone health–related outcomes
S	Randomized control trials or control trails	Qualitative studies, systematic reviews, meta-analyses, study protocols, gray literature, or conference abstracts

Note: P, participants; I, intervention; C, control; O, outcome; S, study design.

**Table 2:** Subgroup analyses based on meta-analyses results of BMD.

Subgroup	K(N)	Hedges’g	95%CI	T-Value	P_d_	I^2^-2	I^2^-3	Power	P_b_
Menopause Stage									**<0.01**
Perimenopause	34 (1616)	0.53	[0.35, 0.71]	5.97	**<0.01**	0%	49.62%	99%	
Postmenopause	44 (2716)	0.22	[0.06, 0.37]	2.83	**0.01**	30.16%	0%	83%	
Body Part									**0.03**
Calcanei	1 (52)	0.25	[−0.39, 0.89]	n/a	n/a	n/a	n/a	n/a	
Femoral neck	11 (682)	0.37	[0.14, 0.59]	3.30	**<0.01**	72.44%	0%	92%	
Greater trochanter	10 (613)	0.33	[0.10, 0.56]	2.90	**<0.01**	41.19%	0%	85%	
Pelvis	2 (82)	−0.01	[−0.51, 0.50]	−0.03	0.98	13.40%	13.4%	5%	
Spine L2–L4	23 (1280)	0.47	[0.28, 0.65]	4.94	**<0.01**	51.99%	0%	99%	
Thigh	5 (323)	0.32	[0.04, 0.60]	2.27	**0.03**	9.83%	0%	63%	
Tibia	4 (124)	0.33	[−0.16, 0.82]	1.34	0.18	0%	0%	28%	
Trunk	2 (82)	0.96	[0.42, 1.49]	3.56	**<0.01**	0%	0%	94%	
Upper limb	9 (502)	0.29	[0.03, 0.56]	2.11	**0.03**	3.64%	0%	61%	
Ward’s triangle	9 (510)	0.27	[0.03, 0.51]	2.23	**0.03**	21.75%	0%	61%	
Whole body	2 (82)	−0.32	[−0.83, 0.19]	−1.23	0.22	40.91%	40.91%	23%	
Taichi Type									0.12
Tai Chi chuan	55 (3472)	0.27	[0.11, 0.43]	3.45	**<0.01**	37.52%	0%	93%	
Tai Chi Push Hands	5 (128)	0.33	[−0.08, 0.74]	1.59	0.12	0%	0%	36%	
Taichi rouli ball	18 (732)	0.60	[0.33, 0.87]	4.42	**<0.01**	0%	63.33%	99%	

Note: Subgroup analyses based on meta-analyses results of BMD. K(N), number of effect size (sample size); Hedges’ g, standardized mean difference; CI, confidence interval; P_d_, *p* value for subgroup differences; P_b_, *p* value between subgroups; I^2^-2, heterogeneity within studies; I^2^-3, heterogeneity between studies; Power, statistical power. Significant results (*p* < 0.05) are in bold.

**Table 3:** Meta interaction Regression plot for body part and menopausal stage in bone mineral density.

Body Part	Menopausal Stage	Predicted SMD	95%CI	*p*-Value
Upper limb	Perimenopause	0.26	[−0.24, 0.77]	0.31
	Postmenopause	0.20	[−0.07, 0.48]	0.15
Ward triangle	Perimenopause	0.43	[0.11, 0.75]	0.01
	Postmenopause	0.18	[−0.12, 0.49]	0.25
Greater trochanter	Perimenopause	0.61	[0.28, 0.93]	<0.01
	Postmenopause	0.15	[−0.12, 0.41]	0.28
Femoral neck	Perimenopause	0.50	[0.17, 0.82]	<0.01
	Postmenopause	0.28	[0.03, 0.53]	0.03
Spine L2–L4	Perimenopause	0.81	[0.54, 1.08]	<0.01
	Postmenopause	0.25	[0.05, 0.46]	0.02

Note: This table summarizes the results of meta-regression analysis for bone mineral density (BMD) across different body parts (upper limb, Ward’s triangle, greater trochanter, femoral neck, and lumbar spine L2–L4) in perimenopausal and postmenopausal women. *p*-value < 0.05 was considered statistically significant and *p* < 0.01 indicated a highly significant difference.

**Table 4:** Subgroup analyses based on meta-analyses results of BMC.

Subgroup	K(N)	Hedges’g	95%CI	T-Value	P_d_	I^2^-2	I^2^-3	Power	P_b_
Body Part									0.58
Hip	3 (120)	2.60	[0.25, 4.94]	2.44	**0.03**	0%	96.57%	69%	
Pelvis	2 (82)	0.79	[−1.51, 3.08]	0.75	0.47	0%	0%	12%	
Spine L2–L4	5 (202)	2.00	[0.11, 3.89]	2.33	**0.04**	94.75%	0%	64%	
Thigh	2 (82)	0.56	[−1.73, 2.85]	0.54	0.60	0%	0%	8%	
Trunk	2 (82)	1.32	[−0.98, 3.63]	1.26	0.23	0%	0%	24%	
upper limb	2 (82)	2.00	[−0.33, 4.33]	1.89	0.09	0%	0%	47%	
whole body	2 (82)	0.57	[−1.72, 2.87]	0.55	0.59	0%	0%	8%	
Duration									0.53
60 min	6 (312)	1.95	[0.84, 3.07]	3.72	**<0.01**	0%	87.81%	96%	
90 min	12 (420)	1.54	[0.74, 2.34]	4.08	**<0.01**	0%	91.08%	98%	
Frequency									0.81
<4 times per week	12 (180)	1.63	[0.82, 2.43]	4.29	**<0.01**	0%	93.85%	99%	
>4 times per week	6 (552)	1.79	[0.64, 2.94]	3.29	**<0.01**	0%	50.63%	91%	

Note: Subgroup analyses based on meta-analyses results of BMC. K(N), number of effect size (sample size); Hedges’ g, standardized mean difference; CI, confidence interval; P_d_, *p* value for subgroup differences; P_b_, *p* value between subgroups; I^2^-2, heterogeneity within studies; I^2^-3, heterogeneity between studies; Power, statistical power. Significant results (*p* < 0.05) are in bold.

**Table 5:** Subgroup analyses based on meta-analyses results of BMM.

Subgroup	K(N)	Hedges’g	95%CI	T-Value	P_d_	I^2^-2	I^2^-3	Power	P_b_
Menopause Stage									0.53
Perimenopause	6 (246)	−4.79	[−15.63, 6.05]	−1.08	0.32	90.80%	8.22%	19%	
Postmenopause	2 (78)	−0.48	[−11.86, 10.90]	−0.10	0.92	36.81%	36.81%	5%	
Duration									0.46
60 min	5 (234)	−4.20	[−13.01, 4.62]	−1.16	0.29	69.08%	29.02%	21%	
90 min	3 (90)	1.28	[−13.42, 15.98]	0.21	0.84	0%	72.58%	6%	
Frequency									0.32
<4 times per week	4 (116)	−5.80	[−15.43, 3.83]	−1.47	0.19	76.43%	21.99%	31%	
>4 times per week	4 (208)	0.23	[−9.38, 9.84]	0.06	0.95	77.23%	16.34%	5%	

Note: Subgroup analyses based on meta-analyses results of BMM. K(N), number of effect size (sample size); Hedges’ g, standardized mean difference; CI, confidence interval; P_d_, *p* value for subgroup differences; P_b_, *p* value between subgroups; I^2^-2, heterogeneity within studies; I^2^-3, heterogeneity between studies; Power, statistical power. Significant results (*p* < 0.05) are in bold.

**Table 6:** Subgroup analyses based on meta-analyses results of BTMs.

Subgroup	K(N)	Hedges’g	95%CI	T-Value	P_d_	I^2^-2	I^2^-3	Power	P_b_
Menopause Stage									0.92
Perimenopause	4 (162)	−0.14	[−0.53, 0.24]	−0.83	0.43	0%	0%	22%	
Postmenopause	8 (982)	−0.12	[−0.35, 0.10]	−1.22	0.25	32.69%	1.31%	13%	
Duration									0.78
<45 min	4 (826)	−0.11	[−0.39, 0.18]	−0.84	0.42	27.46%	5.27%	13%	
>45 min	8 (318)	−0.16	[−0.45, 0.13]	−1.23	0.25	16.02%	0.00%	23%	
Period									0.07
<1 year	8 (352)	−0.27	[−0.52, −0.03]	−2.51	**0.03**	0%	0%	71%	
>1 year	4 (792)	−0.01	[−0.16, 0.15]	−0.06	0.96	0%	5%	5%	
Frequency									0.28
<4 times per week	5 (236)	−0.04	[−0.35, 0.28]	−0.26	0.80	0%	0%	5%	
=4 times per week	4 (826)	−0.08	[−0.30, 0.13]	−0.89	0.40	27.46%	5.27%	14%	
>4 times per week	3 (82)	−0.49	[−1.02, 0.05]	−2.06	0.07	16.21%	0.00%	54%	

Note: Subgroup analyses based on meta-analyses results of BTMs. K(N), number of effect size (sample size); Hedges’ g, standardized mean difference; CI, confidence interval; P_d_, *p* value for subgroup differences; P_b_, *p* value between subgroups; I^2^-2, heterogeneity within studies; I^2^-3, heterogeneity between studies; Power, statistical power. Significant results (*p* < 0.05) are in bold.

**Table 7:** Evaluation of the PEDro scale of the included studies (Median = 6, Interquartile range = 1) [15,16,17,50,51,52,53,54,55,56,57,58,59,60,61,62].

Author Year	D1	D2	D3	D4	D5	D6	D7	D8	D9	D10	D11	Total
Xin 2024 [50]	Y	1	0	1	0	0	0	1	1	1	1	6
Qin 2002 [51]	Y	0	0	1	0	0	0	1	1	1	1	5
Wayne 2012 [52]	Y	1	1	1	0	0	1	1	1	1	1	9
Xiao 2015 [16]	Y	1	0	1	0	0	0	1	1	1	1	6
Cheng 2022 [15]	Y	1	0	1	0	0	0	1	1	1	1	6
Chan 2004 [17]	Y	1	0	1	0	0	1	0	1	1	1	6
Zhou 2003 [53]	Y	1	0	1	0	0	1	1	1	1	1	7
Zhou 2004 [62]	Y	1	0	1	0	0	1	1	1	1	1	7
Zhou 2005 [55]	Y	1	0	1	0	0	1	1	1	1	1	7
Zhao 2020 [54]	Y	1	0	1	0	0	1	1	1	1	1	7
Du 2014 [56]	Y	1	0	1	0	0	0	1	1	1	1	6
Liu 2021 [57]	Y	1	0	1	0	0	0	1	1	1	1	6
Guo 2014 [58]	Y	0	0	1	0	0	0	1	1	1	1	5
Mao 2009 [59]	Y	1	0	1	0	0	0	1	1	1	1	6
Xue 2015 [60]	Y	1	0	1	0	0	0	1	1	1	1	6
Xu 2017 [61]	Y	1	0	1	0	0	0	1	1	1	1	6

Note: studies scoring ≥ 6 are considered high quality, those scoring 4–5 are considered moderate quality, and those scoring ≤ 3 are considered low quality. (1) Eligibility criteria were specified (not included in the total score). (2) Subjects were randomly allocated to groups (in a crossover study, subjects were randomly allocated an order in which treatments were received). (3) Allocation was concealed. (4) The groups were similar at baseline regarding the most important prognostic indicators. (5) There was blinding of all subjects. (6) There was blinding of all therapists who administered the therapy. (7) There was blinding of all assessors who measured at least one key outcome. (8) Measures of at least one key outcome were obtained from more than 85% of the subjects initially allocated to groups. (9) All subjects for whom outcome measures were available received the treatment or control condition as allocated or, where this was not the case, data for at least one key outcome was analyzed by “intention to treat”. (10) The results of between-group statistical comparisons are reported for at least one key outcome. (11) The study provides both point measures and measures of variability for at least one key outcome.

**Table 8:** GRADE assessment results.

Outcome	No. of Participants (Studies)	Certainty Assessment						Effect Size (SMD [95% CI])	Certainty (GRADE)
		Risk of Bias	Inconsistency	Indirectness	Imprecision	Publication Bias	Other		
Bone Mineral Density	700 (14)	Serious	Not serious	Not serious	Not serious	Not serious	None	0.31 [0.16, 0.45]	Moderate
Bone Mineral Content	122 (3)	Serious	Serious	Not serious	Not serious	Not serious	Large Effect Size	1.63 [0.64, 2.62]	Low
Bone Mineral Metabolism	160 (4)	Serious	Serious	Not serious	Not serious	Not serious	Large Effect Size	−2.71 [−12.00, −6.59]	Low
Bone Turnover Markers	613 (7)	Serious	Serious	Not serious	Not serious	Not serious	None	−0.10 [−0.35, 0.16]	Low

Note: The certainty of evidence according to the Grading of Recommendations, Assessment, Development, and Evaluations (GRADE) system is categorized into four levels: high, moderate, low, and very low. High certainty means we are very confident in the estimated effect, while moderate certainty indicates moderate confidence. Low certainty reflects limited confidence in the estimated effect, and very low certainty indicates very little confidence. This classification aids in assessing the reliability of research findings and guides the interpretation and application of evidence.

**Figure A1:**
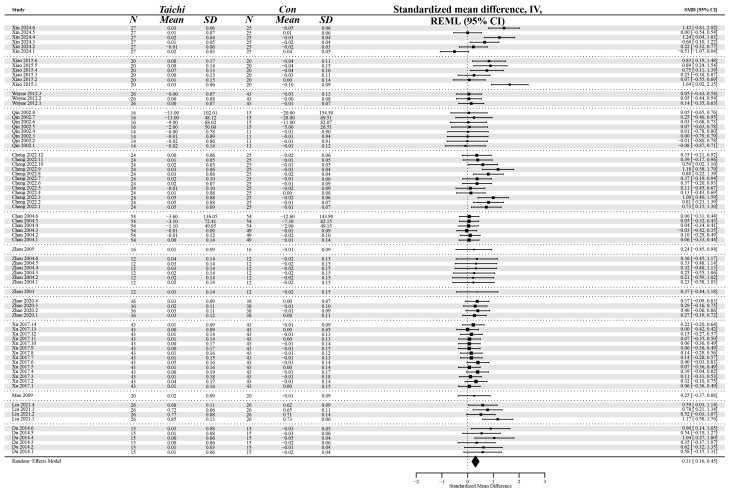
BMD Forest Plot [15,16,17,50,51,52,53,54,55,56,57,59,61,62].

**Figure A2:**
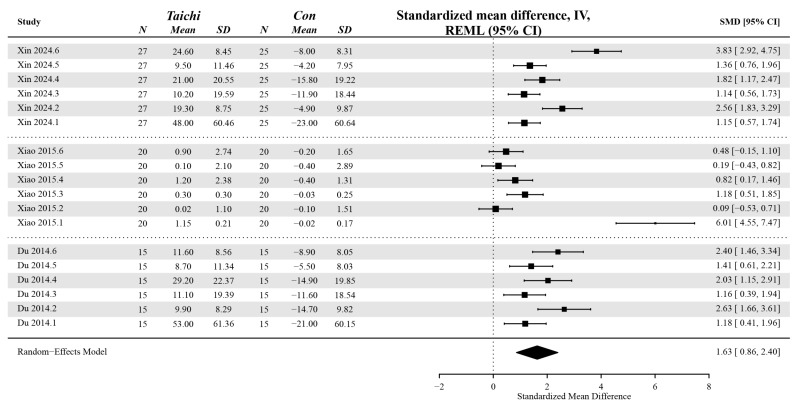
BMC Forest Plot [16,50,56].

**Figure A3:**
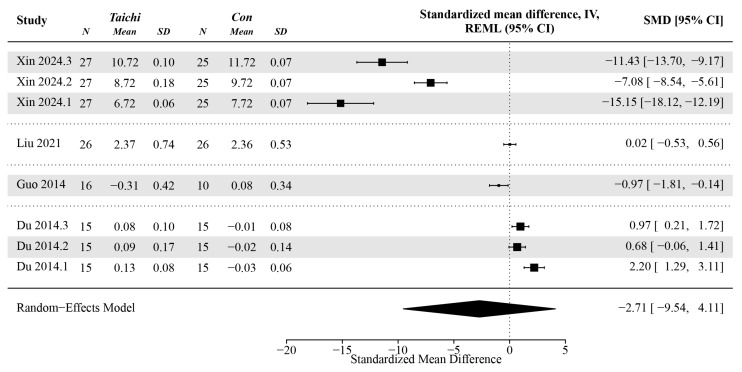
BMM Forest Plot [50,56,57,58].

**Figure A4:**
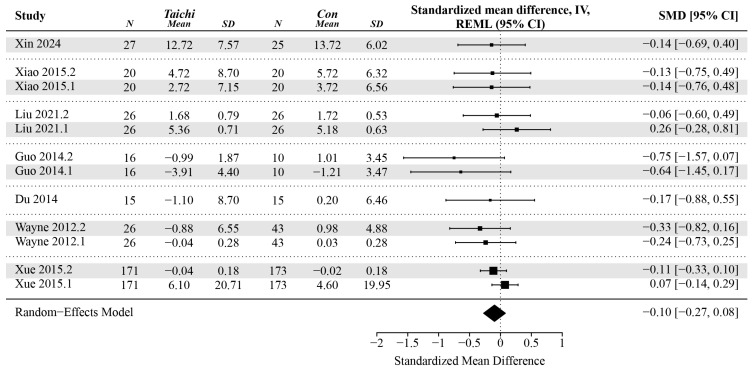
BTMs Forest Plot [16,50,52,56,57,58,60].

**Figure A5:**
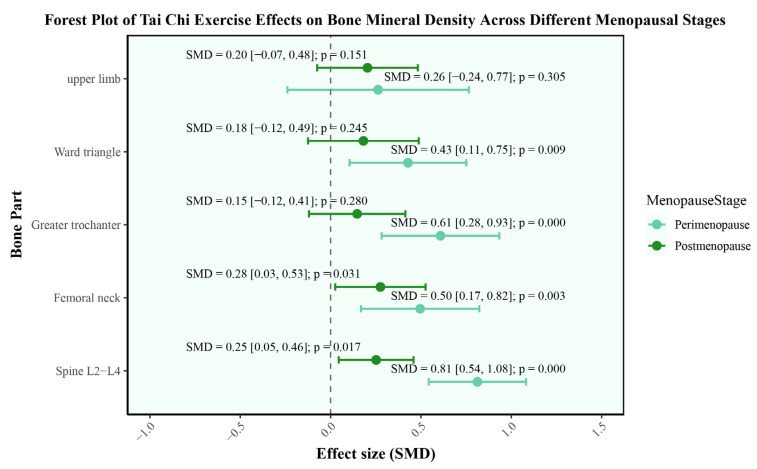
Meta Interaction Regression Plot.

**Figure A6:**
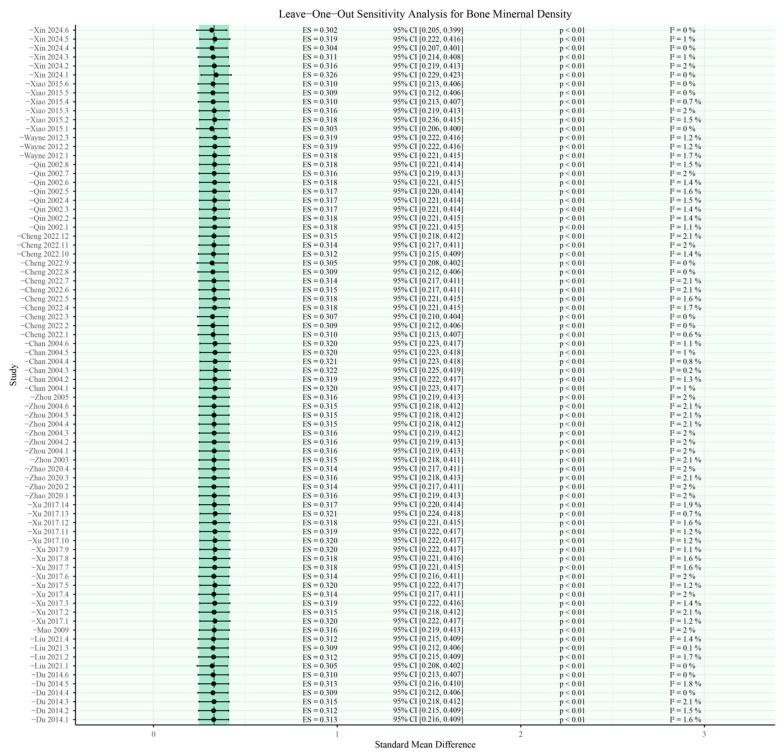
BMD Sensitivity Analysis Plot [15,16,17,50,51,52,53,54,55,56,57,59,61,62].

**Figure A7:**
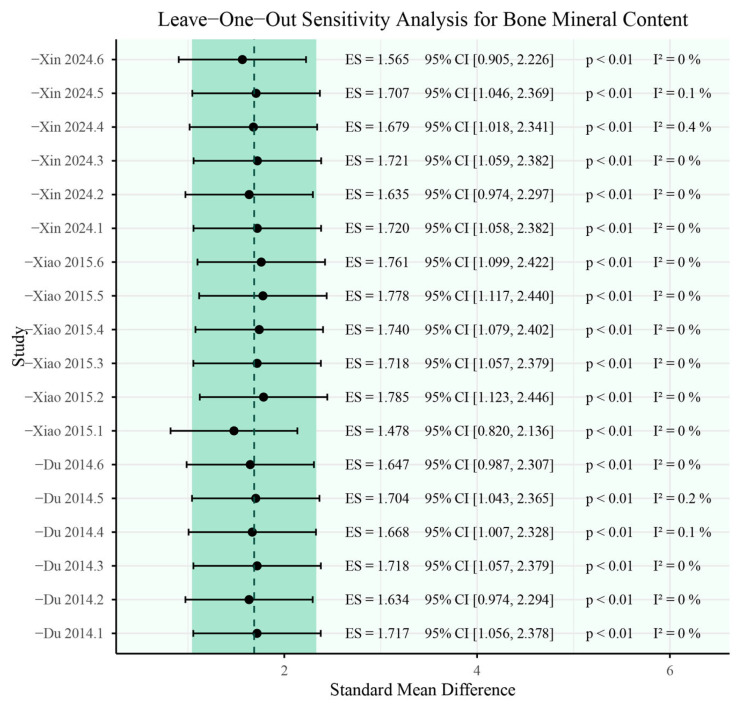
BMC Sensitivity Analysis Plot [16,50,56].

**Figure A8:**
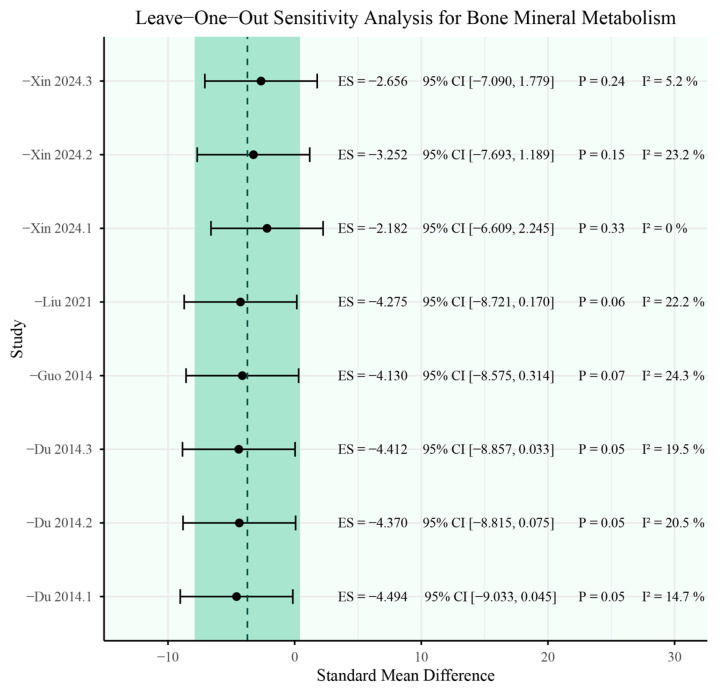
BMM Sensitivity Analysis Plot [50,56,57,58].

**Figure A9:**
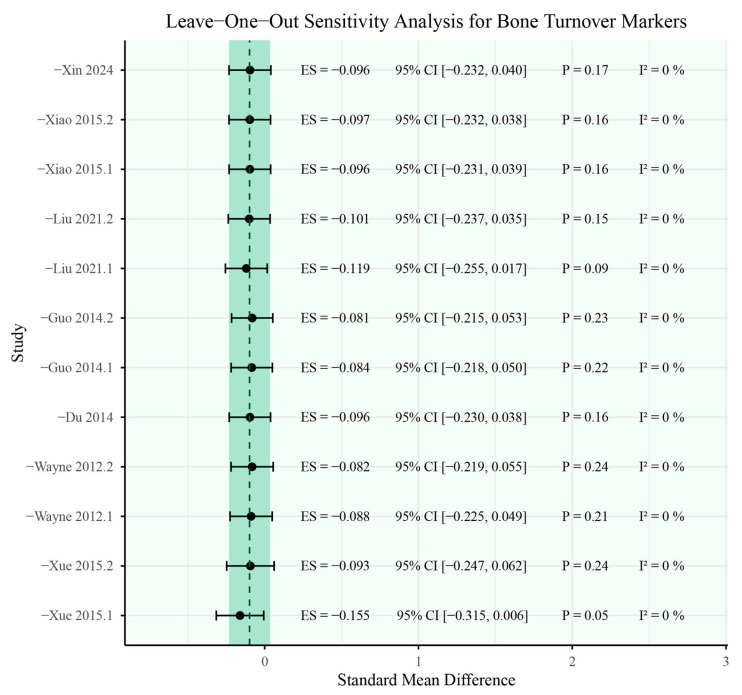
BMM Sensitivity Analysis Plot [16,50,52,56,57,58,60].

## Data Availability

The corresponding author of this article will unconditionally provide all the original data supporting the results of this study.

## Appendix A

**Table A1:** The PRISMA Checklist 2020.

Section and Topic	Item	Checklist Item	Location Where Item Is Reported
Title			
Title	1	Identify the report as a systematic review.	1
Abstract			
Abstract	2	See the PRISMA 2020 for Abstracts checklist.	1
Introduction			
Rationale	3	Describe the rationale for the review in the context of existing knowledge.	2
Objectives	4	Provide an explicit statement of the objective(s) or question(s) the review addresses.	3
Methods			
Eligibility criteria	5	Specify the inclusion and exclusion criteria for the review and how studies were grouped for the syntheses.	3, 4
Information sources	6	Specify all databases, registers, websites, organizations, reference lists and other sources searched or consulted to identify studies. Specify the date when each source was last searched or consulted.	3
Search strategy	7	Present the full search strategies for all databases, registers and websites, including any filters and limits used.	3
Selection process	8	Specify the methods used to decide whether a study met the inclusion criteria of the review, including how many reviewers screened each record and each report retrieved, whether they worked independently, and if applicable, details of automation tools used in the process.	3
Data collection process	9	Specify the methods used to collect data from reports, including how many reviewers collected data from each report, whether they worked independently, any processes for obtaining or confirming data from study investigators, and if applicable, details of automation tools used in the process.	4, 5
Data items	10a	List and define all outcomes for which data were sought. Specify whether all results that were compatible with each outcome domain in each study were sought (e.g., for all measures, timepoints, analyses), and if not, the methods used to decide which results to collect.	4
	10b	List and define all other variables for which data were sought (e.g., participant and intervention characteristics, funding sources). Describe any assumptions made about any missing or unclear information.	4
Study risk of bias assessment	11	Specify the methods used to assess risk of bias in the included studies, including details of the tool(s) used, how many reviewers assessed each study and whether they worked independently, and if applicable, details of automation tools used in the process.	5, 6
Effect measures	12	Specify for each outcome the effect measure(s) (e.g., risk ratio, mean difference) used in the synthesis or presentation of results.	6
Synthesis methods	13a	Describe the processes used to decide which studies were eligible for each synthesis (e.g., tabulating the study intervention characteristics and comparing against the planned groups for each synthesis (item #5)).	6
	13b	Describe any methods required to prepare the data for presentation or synthesis, such as handling of missing summary statistics, or data conversions.	6
	13c	Describe any methods used to tabulate or visually display results of individual studies and syntheses.	6
	13d	Describe any methods used to synthesize results and provide a rationale for the choice(s). If meta-analysis was performed, describe the model(s), method(s) to identify the presence and extent of statistical heterogeneity, and software package(s) used.	6
	13e	Describe any methods used to explore possible causes of heterogeneity among study results (e.g., subgroup analysis, meta-regression).	6
	13f	Describe any sensitivity analyses conducted to assess robustness of the synthesized results.	6
Reporting bias assessment	14	Describe any methods used to assess risk of bias due to missing results in a synthesis (arising from reporting biases).	5, 6
Certainty assessment	15	Describe any methods used to assess certainty (or confidence) in the body of evidence for an outcome.	6, 7
Results			
Study selection	16a	Describe the results of the search and selection process, from the number of records identified in the search to the number of studies included in the review, ideally using a flow diagram.	7
	16b	Cite studies that might appear to meet the inclusion criteria, but which were excluded, and explain why they were excluded.	n/a
Study characteristics	17	Cite each included study and present its characteristics.	8, 42, 43, 44, 45, 46
Risk of bias in studies	18	Present assessments of risk of bias for each included study.	17, 18
Results of individual studies	19	For all outcomes, present, for each study: (a) summary statistics for each group (where appropriate) and (b) an effect estimate and its precision (e.g., confidence/credible interval), ideally using structured tables or plots.	8, 9, 10
Results of syntheses	20a	For each synthesis, briefly summarize the characteristics and risk of bias among contributing studies.	8, 9, 10
	20b	Present results of all statistical syntheses conducted. If meta-analysis was performed, present for each the summary estimate and its precision (e.g., confidence/credible interval) and measures of statistical heterogeneity. If comparing groups, describe the direction of the effect.	8, 9, 10
	20c	Present results of all investigations of possible causes of heterogeneity among study results.	10, 11, 12, 13, 14, 15, 16, 17
	20d	Present results of all sensitivity analyses conducted to assess the robustness of the synthesized results.	17
Reporting biases	21	Present assessments of risk of bias due to missing results (arising from reporting biases) for each synthesis assessed.	17, 18
Certainty of evidence	22	Present assessments of certainty (or confidence) in the body of evidence for each outcome assessed.	19
Discussion			
Discussion	23a	Provide a general interpretation of the results in the context of other evidence.	20
	23b	Discuss any limitations of the evidence included in the review.	20, 21, 22, 23, 24, 25, 26, 27, 28
	23c	Discuss any limitations of the review processes used.	20, 21, 22, 23, 24, 25, 26, 27, 28
	23d	Discuss implications of the results for practice, policy, and future research.	21, 22, 23
Other Information			
Registration and protocol	24a	Provide registration information for the review, including register name and registration number, or state that the review was not registered.	3
	24b	Indicate where the review protocol can be accessed, or state that a protocol was not prepared.	3
	24c	Describe and explain any amendments to information provided at registration or in the protocol.	n/a
Support	25	Describe sources of financial or non-financial support for the review, and the role of the funders or sponsors in the review.	28
Competing interests	26	Declare any competing interests of review authors.	28
Availability of data, code and other materials	27	Report which of the following are publicly available and where they can be found: template data collection forms; data extracted from included studies; data used for all analyses; analytic code; any other materials used in the review.	n/a

Note: n/a, not available.

## Appendix B

**Table A2:** Detailed Search Strategy.

Database	Search Strategy	Number of Articles
PubMed	((tai chi) OR(taiji) OR(tai ji quan) OR(tai-chi) OR(tai chi chuan) OR(shadow boxing)) AND((menopause) OR(menopausal women) OR(postmenopausal women) OR(middle-aged women) OR(climacteric)) AND((bone mineral density) OR(BMD) OR(osteoporosis) OR(bone health) OR(bone loss) OR(bone metabolism) OR(bone turnover) OR(bone fragility) OR(skeletal health) OR(bone remodeling) OR(bone strength))	72
Web of science	((TI = tai chi) OR(TI = taiji) OR(TI = tai ji quan) OR(TI = tai-chi) OR(TI = tai chi chuan) OR(TI = shadow boxing)) AND((AB = menopause) OR(AB = menopausal women) OR(AB = postmenopausal women) OR(AB = middle-aged women) OR(AB = climacteric)) AND((AB = bone mineral density) OR(AB = BMD) OR(AB = osteoporosis) OR(AB = bone health) OR(AB = bone loss) OR(AB = bone metabolism) OR(AB = bone turnover) OR(AB = bone fragility) OR(AB = skeletal health) OR(AB = bone remodeling) OR(AB = bone strength))	36
Cochrane library	#1((tai chi) OR(taiji) OR(tai ji quan) OR(tai-chi) OR(tai chi chuan) OR(shadow boxing)):ti,ab,kw #2((menopause) OR(menopausal women) OR(postmenopausal women) OR(middle-aged women) OR(climacteric)):ti,ab,kw #3((bone mineral density) OR(BMD) OR(osteoporosis) OR(bone health) OR(bone loss) OR(bone metabolism) OR(bone turnover) OR(bone fragility) OR(skeletal health) OR(bone remodeling) OR(bone strength)):ti,ab,kw #4: #1 AND #2 AND#3	38
Embase	#1((tai chi) OR(taiji) OR(tai ji quan) OR(tai-chi) OR(tai chi chuan) OR(shadow boxing)):ti,ab,kw #2((menopause) OR(menopausal women) OR(postmenopausal women) OR(middle-aged women) OR(climacteric)):ti,ab,kw #3((bone mineral density) OR(BMD) OR(osteoporosis) OR(bone health) OR(bone loss) OR(bone metabolism) OR(bone turnover) OR(bone fragility) OR(skeletal health) OR(bone remodeling) OR(bone strength)):ti,ab,kw #4: #1 AND #2 AND#3	56
SPORTDiscus (Via EBSCO)	TX ((tai chi) OR(taiji) OR(tai ji quan) OR(tai-chi) OR(tai chi chuan) OR(shadow boxing)) AND((menopause) OR(menopausal women) OR(postmenopausal women) OR(middle-aged women) OR(climacteric)) AND((bone mineral density) OR(BMD) OR(osteoporosis) OR(bone health) OR(bone loss) OR(bone metabolism) OR(bone turnover) OR(bone fragility) OR(skeletal health) OR(bone remodeling) OR(bone strength))	21
MEDLINE (Via EBSCO),	TX ((tai chi) OR(taiji) OR(tai ji quan) OR(tai-chi) OR(tai chi chuan) OR(shadow boxing)) AND((menopause) OR(menopausal women) OR(postmenopausal women) OR(middle-aged women) OR(climacteric)) AND((bone mineral density) OR(BMD) OR(osteoporosis) OR(bone health) OR(bone loss) OR(bone metabolism) OR(bone turnover) OR(bone fragility) OR(skeletal health) OR(bone remodeling) OR(bone strength))	50
CNKI	SU = (‘太极’ + ‘太极拳’ + ‘太极运动’) and SU = (‘更年期女性’ + ‘更年期’ + ‘围绝经期’ + ‘绝经后女性’ + ‘中年女性’) and SU = (‘骨密度’ + ‘骨质疏松’ + ‘骨骼健康’ + ‘骨丢失’ + ‘骨代谢’ + ‘骨强度’ + ‘骨质重建’ + ‘骨骼脆性’ + ‘骨转化标志物’)	20
WanFang	题名或关键词:((太极拳) OR(太极) OR(太极运动)) AND 题名或关键词:((更年期女性) OR(更年期) OR(围绝经期) OR(绝经后女性) OR(中年女性)) AND 题名或关键词:((骨密度) OR(骨质疏松) OR(骨骼健康) OR(骨丢失) OR(骨代谢) OR(骨强度) OR(骨质重建) OR(骨骼脆性) OR(骨转化标志物))	21
VIP	((M = 太极拳 OR M = 太极 OR M = 太极运动)) AND ((M = 更年期女性 OR M = 更年期 OR M = 围绝经期 OR M = 绝经后女性 OR M = 中年女性)) AND ((M = 骨密度 OR M = 骨质疏松 OR M = 骨骼健康 OR M = 骨丢失 OR M = 骨代谢) OR M = 骨强度 OR M = 骨质重建 OR M = 骨骼脆性 OR M = 骨转化标志物))	59

Note: CNKI, China National Knowledge Infrastructure; VIP, Database for Chinese Technical Periodicals. The Chinese search strategies were used for Chinese databases (e.g., CNKI, WanFang and VIP).

## Appendix C

**Table A3:** The Characteristics for the Studies Included [15,16,17,50,51,52,53,54,55,56,57,58,59,60,61,62].

First Author	Design	Participants	Terms	Menopausal Stage	Tai Chi Sessions	Con Sessions	Dur (min)	Fre	Wk	PEDro
Du, 2014 [56]	RCT	Tai Chi—N: 15 Con—N: 15 Tai Chi—Age: 51.69 ± 3.18 Con—Age: 52.21 ± 3.01	Tai Chi Rouli ball	Perimenopause	Tai Chi Rouli balll Routine 1–3 Intensity: Heart rate 110–130 beats/min.	The control group did not exercise and maintained their original lifestyle.	90	4.5	24	6
Guo, 2014 [58]	RCT	Tai Chi—N: 16 Con—N: 10 Tai Chi—Age: 56.69 ± 3.50 Con—Age: 56.60 ± 4.30	Tai Chi Quan	Postmenopause	24-form Tai Chi Chuan Intensity: 50% HR_max_ at beginning; 60–80% HR_max_ in the whole process.	Con group engaged in daily physical activities, and no one withdrew midway throughout the entire process.	60	6	24	5
Mao, 2009 [59]	RCT	Tai Chi—N: 20 Con—N: 20 Age: 56.78 ± 2.90	Tai Chi Quan	Postmenopause	Prepare the activity time for 10 min, organize the activity time for 5–10 min, and formally engage in at least 30 min of moderate intensity Tai Chi exercise. Intensity: Heart rate 110 beats/min.	No intervention is made, the control group maintained their original lifestyle and received regular health education.	30	NR	20	6
Liu, 2021 [57]	RCT	Tai Chi—N: 26 Con—N: 26 Age: 56.48 ± 3.41	Tai Chi Quan	Postmenopause	Adopting 24 simplified Tai Chi exercises, guided by professional Tai Chi coaches, strictly following the requirements of Tai Chi training during practice, emphasizing on techniques, moves, and thoughts. Intensity: The standard heart rate during exercise is “170-age”.	The subjects did not engage in long-term exercise of Tai Chi, but some individuals occasionally exercised	60	3	52	6
Xu, 2017 [61]	RCT	Tai Chi—N: 43 Con—N: 43 Tai Chi—Age: 54–65 Con—Age: 53–67	Tai Chi Quan	Postmenopause	Tai Chi group practice 24 simplified Tai Chi exercises.	Con group engaged in daily physical activities, and no one withdrew midway throughout the entire process.	40	6	52	6
Xue, 2015 [60]	RCT	Tai Chi—N: 171 Con—N: 173 Tai Chi—Age: 62.1 ± 7.0 Con—Age: 64.0 ± 7.3	Tai Chi Quan	Postmenopause	Regular health education is provided for the Tai Chi group, using a unified arrangement of bone health exercise content (Tai Chi) for training. Participants should participate in the exercise program in an organized and regular manner and record it.	The control group maintained their original lifestyle and received regular health education.	30	4	104	6
Zhao, 2020 [54]	RCT	Tai Chi—N: 36 Con—N: 38 Tai Chi—Age: 49.7 ± 3.9 Con—Age: 49.7 ± 3.9	Tai Chi Quan	Perimenopause	Under the guidance of a professional Tai Chi coach (group teaching), a 48 week 24 style simplified Tai Chi intervention will be conducted. Intensity: The subject’s heart rate should be controlled between 55% and 65% HR_max_. If it exceeds the controlled heart rate, the subject’s exercise intensity should be appropriately reduced.	Do not engage in any other forms of exercise except for maintaining past lifestyle habits.	60	3	48	7
Zhou, 2003 [53]	RCT	Tai Chi—N: 12 Con—N: 10 Tai Chi—Age: 57.10 ± 2.71 Con—Age: 55.96 ± 2.84	Tai Chi Push hands	Postmenopause	In the early stage, the main focus is on fixed step push hand exercises, while in the later stage, the main focus is on active step push hand exercises, with increasing intensity and difficulty.	Not participating in sports activities and maintaining normal lifestyle habits.	52.5	6	43.5	7
Zhou, 2004 [62]	RCT	Tai Chi 1—N: 12 Tai Chi 2—N: 12 Con: 12 Age: 55.94 ± 2.83	Tai Chi 1: Tai Chi Quan Tai Chi 2: Tai Chi Push hands	Postmenopause	The early stage mainly involves high posture training, while the later stage mainly involves low posture training, with increasing intensity and difficulty.	Not participating in sports activities and maintaining normal lifestyle habits.	52.5	6	43.5	7
Zhou, 2005 [55]	RCT	Tai Chi—N: 16 Con—N: 16 Tai Chi—Age: 51.69 ± 3.18 Age: 57.21 ± 3.41	Tai Chi Push hands	Postmenopause	Start with high posture training and gradually reduce body posture; In terms of footwork, perform fixed step push hand training in the early stage, and then gradually transition to active step training.	Not participating in sports activities and maintaining normal lifestyle habits.	52.5	6	43.5	7
Chan, 2004 [17]	RCT	Tai Chi—N: 54 Con—N: 54 Tai Chi—Age: 54.4 ± 3.3 Con—Age: 53.6 ± 3.2	Tai Chi Quan	Postmenopause	Tai Chi group participated in a supervised Tai Chi exercise (Yang style).	Control subjects retained their sedentary lifestyle without participation in physical exercises.	50	4.2	52	6
Cheng, 2022 [15]	RCT	Tai Chi—N: 24 Con—N: 25 Tai Chi—Age: 50.2 ± 3.1 Con—Age: 50.1 ± 2.9	Tai Chi Quan	Perimenopause	A 24-style simplified form of TC was selected for the TC group. The TC exercises have a fixed voice prompt, including 24 movements (∼5 min is consumed to complete the exercise), and each movement completion time is fixed, for which the exercise rate is fixed.	The cadence of the BW group was not <90 steps/minute (4.8 km/h). The exercise intensity was based on that used in a previous study	60	3	48	6
Qin, 2002 [51]	CT	Tai Chi—N: 16 Con—N: 15 Tai Chi—Age: 54.1 ± 3.7 Con—Age: 53.8 ± 3.6	Tai Chi Quan	Postmenopause	The Tai Chi Chuan exercisers committed to continue their regular exercise for at least 3.5 h/wk in the 12 months after the baseline measurements and to undergo monitoring of their compliance.	The non-exercising controls did not participate in any physical exercise.	70	3	6	5
Wayne, 2012 [52]	RCT	Tai Chi—N: 26 Con—N: 43 Tai Chi—Age: 60.4 ± 5.3 Con—Age: 59.1 ± 4.9	Tai Chi Quan	Postmenopause	Participants in the TC group received nine months of TC training in addition to usual care. They were asked to practice an additional two times per week during the first month, and three times per week thereafter, which could be home practice or additional classes at their school.	Not participating in sports activities and maintaining normal lifestyle habits.	45	4	39.1	9
Xiao, 2015 [16]	RCT	Tai Chi—N: 20 Con—N: 20 Age: 55.5	Tai Chi Rouli ball	Perimenopause	The Tai Chi group practiced Tai Chi Rouli ball for 6 months.	The control group received no intervention.	90	3	26	6
Xin, 2024 [50]	RCT	Tai Chi—N: 27 Con—N: 25 Tai Chi—Age: 51.15 ± 3.32 Con—Age: 50.93 ± 3.66	Tai Chi Rouli ball	Perimenopause	Participants in the Tai Chi Rouli Ball group engaged in the exercise regimen three times a week for a period of six months. Intensity: moderate (was quantitatively assessed using the Borg Rating of Perceived Exertion scale).	The control group kept weekly self-reported physical activity diaries, which were used to monitor any major changes in their usual routines that might influence the study outcomes.	60	3	26	6

Note: N, sample size; Fre, training frequency (sessions/week); Wk, training intervention weeks; Dur, single exercise duration; RCT, randomized controlled trial; CT, non-randomized controlled trials; NR, not report; CON, control; HR, heart rate.
